# The Smallest Diplodocid Skull Reveals Cranial Ontogeny and Growth-Related Dietary Changes in the Largest Dinosaurs

**DOI:** 10.1038/s41598-018-32620-x

**Published:** 2018-10-11

**Authors:** D. Cary Woodruff, Thomas D. Carr, Glenn W. Storrs, Katja Waskow, John B. Scannella, Klara K. Nordén, John P. Wilson

**Affiliations:** 1 Department of Ecology and Evolutionary Biology, University of Toronto, Toronto, ON Canada; 2 Royal Ontario Museum, University of Toronto, Toronto, ON Canada; 3 Great Plains Dinosaur Museum, Malta, MT USA; 4 Department of Biology, Carthage College, Kenosha, WI USA; 5 Cincinnati Museum Center, Cincinnati, OH USA; 6 Steinmann Institute for Geology, Mineralogy, and Paleontology, University of Bonn, Bonn, Germany; 7 Museum of the Rockies, Montana State University, Bozeman, MT USA; 8 Department of Ecology and Evolutionary Biology, Princeton University, Princeton, NJ USA; 9 Department of Paleontology, Badlands Dinosaur Museum, Dickinson Museum Center, Dickinson, ND USA

## Abstract

Sauropod dinosaurs were the largest terrestrial vertebrates; yet despite a robust global fossil record, the paucity of cranial remains complicates attempts to understand their paleobiology. An assemblage of small diplodocid sauropods from the Upper Jurassic Morrison Formation of Montana, USA, has produced the smallest diplodocid skull yet discovered. The ~24 cm long skull is referred to cf. *Diplodocus* based on the presence of several cranial and vertebral characters. This specimen enhances known features of early diplodocid ontogeny including a short snout with narrow-crowned teeth limited to the anterior portion of the jaws and more spatulate teeth posteriorly. The combination of size plus basal and derived character expression seen here further emphasizes caution when naming new taxa displaying the same, as these may be indicative of immaturity. This young diplodocid reveals that cranial modifications occurred throughout growth, providing evidence for ontogenetic dietary partitioning and recapitulation of ancestral morphologies.

## Introduction

With their titanic bodies and long necks and tails, sauropods are perhaps the most recognizable non-avian dinosaurs. *Diplodocus* is one of the best-known sauropod taxa, represented by over 100 specimens since its discovery in 1878^1^. Whereas the postcrania of *Diplodocus* are well represented, cranial remains are exceedingly rare. An adult *Diplodocus* might attain a body length in excess of 30 m^1^, but its skull was well under 1 m^2^. To date only three of these skulls are hypothesized to be from immature animals^3–5^, thus biasing our understanding of this taxon’s ontogeny, ecology, and evolution towards adult specimens. While few, these immature skulls reveal insights into cranial allometry through ontogeny and suggest that *Diplodocus* and its Diplodocidae kin underwent radical ontogenetic change. Such changes would have significant effects on the ecology of immature Diplodocidae and the life history of these animals.

The smallest of the three immature skulls (CM 11255) is 29.2 cm in length, slightly over half of the adult cranial length^3^. Although this specimen reveals critical ontogenetic components, the cranial attributes of much younger diplodocids have remained unknown. Here we describe a new immature cf. *Diplodocus* skull (CMC VP14128), which, with a total cranial length of ~24 cm, represents the smallest known example. This important new specimen reveals hitherto unknown aspects of immature diplodocid anatomy, and shows that juveniles are not merely smaller versions of adults (*sensu* Whitlock *et al*.^3^). Our primary objectives are to test the taxonomic identity of the specimen using phylogenetic analyses, comparative qualitative and quantitative methods, and discuss the ecological implications of cranial ontogeny in diplodocids. We use immature and mature to refer specifically to developmental history, while juvenile, sub-adult, and adult are used as maturational colloquialisms.

## Material and Methods

### Specimens

**Figure 1:**
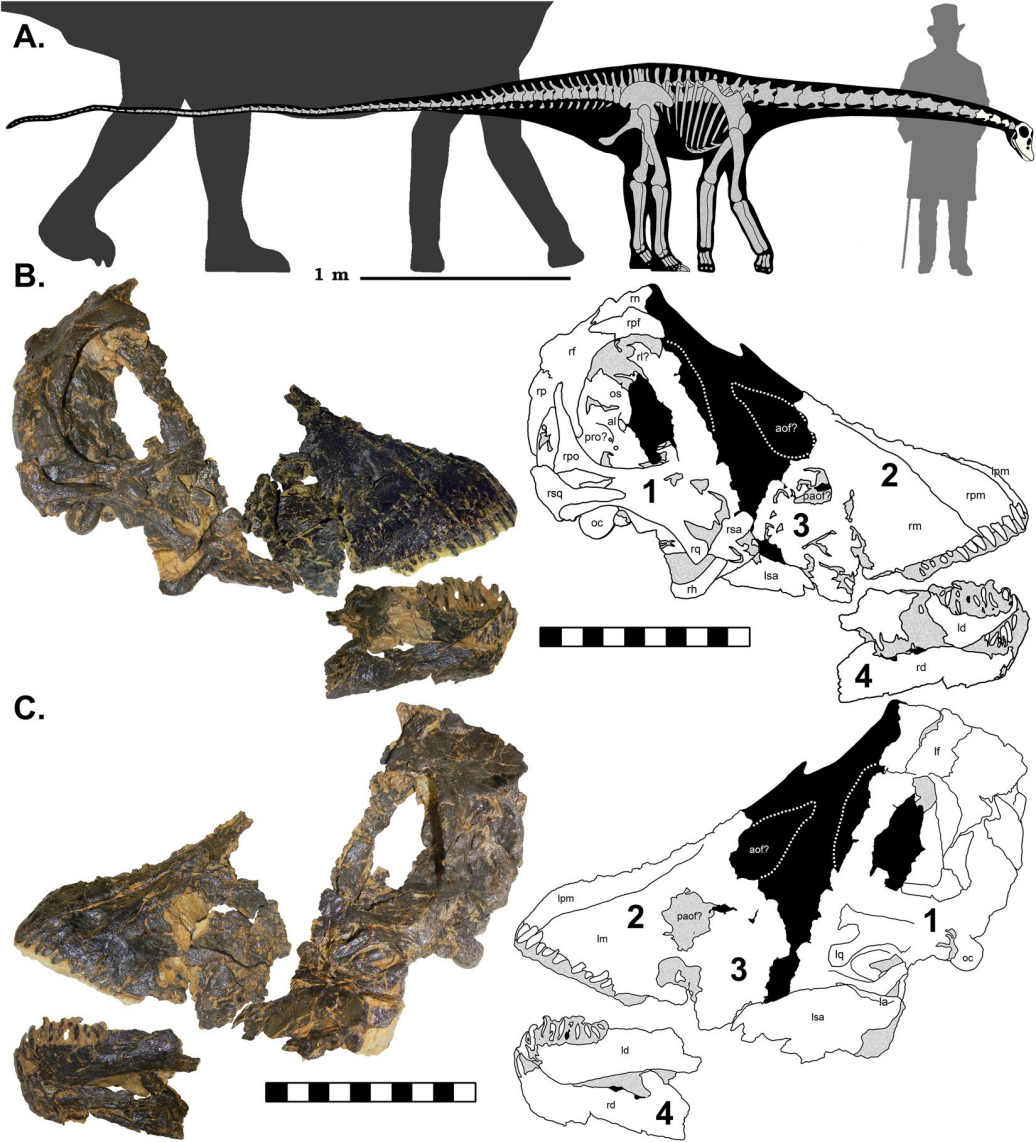
Skeletal reconstruction of CMC VP14128 to scale with a mature *D*. *carnegii* (dark grey). Grey bones are missing, while those in ivory are those present in CMC VP14128. Skeletal reconstruction based on the *Diplodocus* by S. Hartman. Silhouettes by S. Hartman and PhyloPic (Creative Commons Attribution-ShareAlike 3.0 Unported; http://phylopic.org/image/3cb1d5bf-7db5-4db2-82a6-4c39f6a4441b/; https://creativecommons.org/licenses/by-sa/3.0/), modifications made. Skeletal reconstruction of CMC VP14128 redrawn from *D*. *carnegii* skeletal by S. Hartman (http://www.skeletaldrawing.com/sauropods-and-kin/diplodocus). Human scale is Andrew Carnegie at his natural height of 1.6 m. Skeletal and silhouettes to scale. (**B**) CMC VP14128 in right lateral view with accompanying schematic. (**C**) CMC VP14128 in left lateral view with accompanying schematic. Schematics by DCW. The four portions of the skull numbered on accompanying schematics. Lateral views and schematics to scale. a: angular, al: alisphenoid, aof: antorbital fenestra, d: dentary, f: frontal, h: hyoid, l: lacrimal, m: maxilla, n: nasal, oc: occipital condyle, os: orbitosphenoid, p: parietal, paof: preantorbital fenestra, pf: prefrontal, pm: premaxilla, po: postorbital, pro: prootic, q: quadrate, sa: surangular, sq: squamosal. L and r before bone denotes if it is left or right.

CMC VP14128 was collected in 2010 from the Mother’s Day Quarry (MDQ) of south central Montana (MOR locality no. M-166). The MDQ is a monodominant bone bed containing the remains of at least sixteen small diplodocines^6,7^ (recorded femur lengths between 59.5 cm–120 cm^5,8^). While five partial braincases have previously been collected from the MDQ, CMC VP14128 is the only complete skull found at the site. The skull of CMC VP14128 is preserved in four major segments (Fig. 1). CMC VP14128 also preserves one half of the proatlas and at least four anterior cervical vertebrae that were clustered with the cranial remains. A second, similarly sized, isolated, and less distorted braincase (CMC VP14129) was also found in the opposite side of the same field jacket.

### Skull length estimates

Anteroposterior skull length was estimated using a linear regression of skull length to lower jaw length in diplodocid genera (Fig. S3). Scaling the anterior and posterior portions of the lower jaw of the immature *Diplodocus* SMM P84.15.3 results in a total jaw length of 19.9 cm, which indicates approximately 5 cm of missing cranial bone. As the cranial dimensions of smaller (and presumably younger) specimens do not develop isometrically (*sensu* Whitlock *et al*.^3^), from CMC VP14128 it appears that the surangular is proportionally smaller and that most of the lower jaw is represented. This approach produces a conservative estimate of 1 cm of missing bone, which results in a jaw length of 15.6 cm and a skull length of 24.3 cm. This shorter estimate is supported by the preservation of the paired dentaries, posterior portions of the jaw and associated ceratobranchial.

### Transformation grid

**Figure 2:**
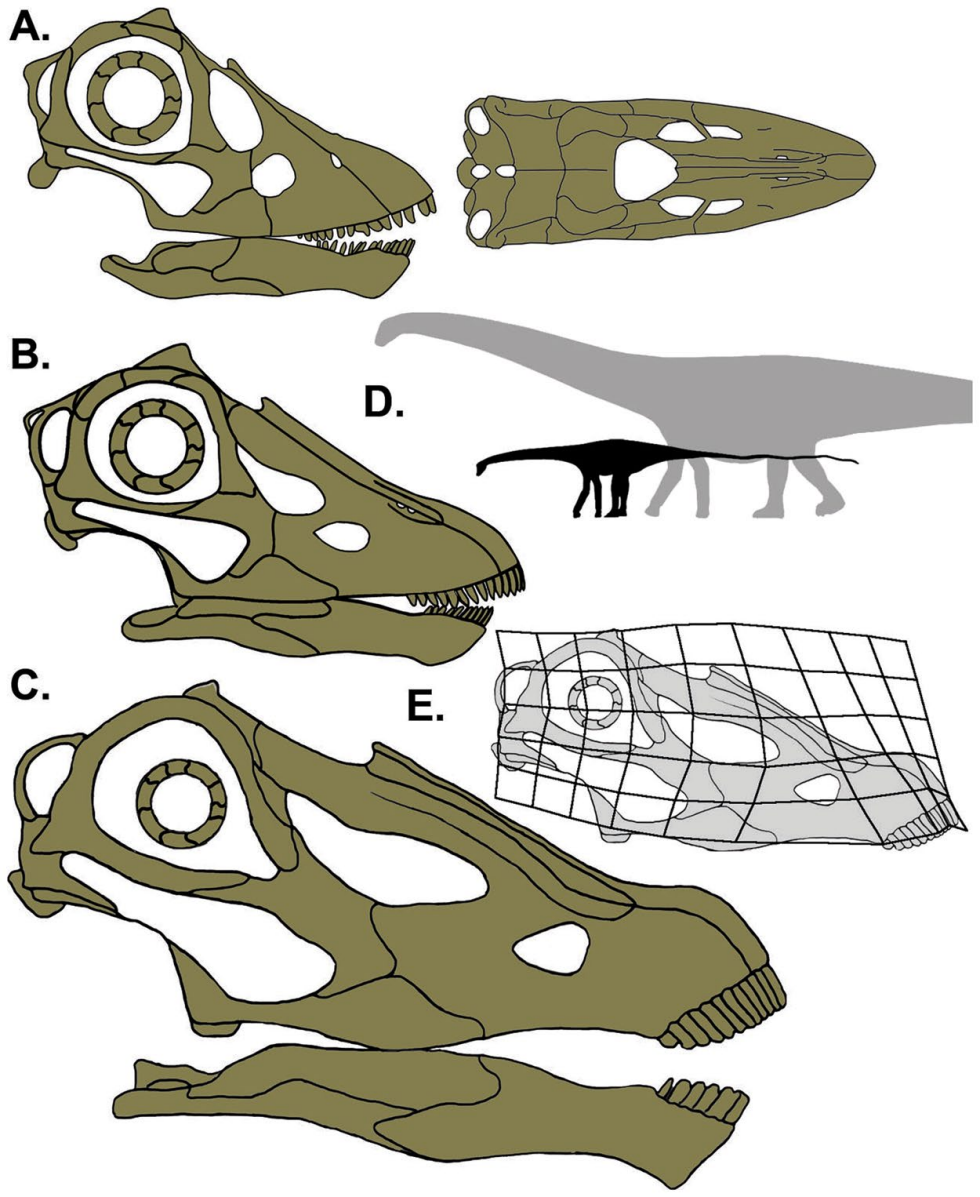
Cranial ontogeny in *Diplodocus* sp. (**A**) CMC VP14128; (**B**) CM 11255 (redrawn from Whitlock *et al*.^3^); (**C**) CM 11161 (redrawn from Wilson and Sereno^69^). Skull drawings by K. Scannella. Skulls to scale. (**D**) Silhouettes of CMC VP14128 and a mature *D*. *carnegii* to illustrate body length differences between skulls of A and C size. *Diplodocus* silhouette by S. Hartman and PhyloPic (Creative Commons Attribution-ShareAlike 3.0 Unported; http://phylopic.org/image/3cb1d5bf-7db5-4db2-82a6-4c39f6a4441b/; https://creativecommons.org/licenses/by-sa/3.0/), modifications made. Skeletal reconstruction of CMC VP14128 redrawn from *D*. *carnegii* skeletal by S. Hartman (http://www.skeletaldrawing.com/sauropods-and-kin/diplodocus). (**E**) Transformation grid highlighting the ontogenetic cranial changes. Adult skull is the same in part C (Wilson and Sereno^69^).

A transformation grid (Fig. 2E) highlighting shape changes between CMC VP14128 and an adult *Diplodocus* was produced using the program tpsSplin^8^. Landmarks were placed on the line drawings of CMC VP14128 and CM 11255 presented in Fig. 2, using the programs tpsUtil^9^ and tpsDig264^10^ (see Fig. S4).

### Phylogenetic analyses

We tested the taxonomic identity of CM VP14128 by including it in the phylogenetic analyses of Whitlock^11^ and Tschopp *et al*.^12^. As CMC VP14128 is predominantly represented by cranial material, in addition to the full analyses incorporating all skeletal characters, we also phylogenetically analyzed CMC VP14128 using only cranial characters. An approach such as thus is certainly not fool-proof, nor should the results be unanimously accepted to represent unequivocal relationships – we simply took this approach to examine what, if any, effects might occur from such extensive missing data. We also combined the matrices (excluding redundant characters) into a single data set – 540 characters for the cranial + postcranial matrix (115 scorable characters – 21% accounted for, 79% missing), and 153 characters for the cranial only matrix (98 scorable characters – 64% accounted for, 36% missing).

Although cranial and vertebral characters identify CMC VP14128 as a diplodocid, it possesses features seen in more inclusive clades, including an extended tooth row, proportionally small antorbital fenestra, and dorsoventrally tall maxilla and premaxilla, which are seen in derived Eusauropoda and basal Macronaria. Paradoxically, if portions of the skull and vertebrae (such as the spatulate teeth and extended tooth row versus the centrum which lack a strong ventral curvature and possess posteriorly elongate postzygapophyses, see below) of CMC VP14128 had been discovered separately, they could have been misidentified.

Furthermore, recent analyses indicate that diplodocids, and potentially all sauropods, underwent allometric growth, and consequently radical ontogenetic trajectories^3,5,13,14^. The documentation of specimens that fill voids in our understanding of sauropod ontogeny is critical. As Rozhdestvensky^15^ noted, derived immature individuals can appear more morphologically similar to basal adults than to their own adult form; thus, this developmental aphorism reveals the multifaceted importance of such specimens.

Within the Morrison Formation, some relatively small body size diplodocids have been used to establish new genera (including *Suuwassea*^16^ and *Kaatedocus*^17^), and the taxonomy of some has changed in different analyses (e.g. *Suuwassea*^11,12,16,18–20^). When examined using phylogenetic analyses, while there is more recent taxonomic consensus (again *Suuwassea*^11,12,20^) some previous analyses placed such taxa in more basal positions^18,19^. A similar phenomenon has also been seen in other dinosaurian clades^21–26^. Therefore, CMC VP14128 present the opportunity to assess the phylogenetic position of a demonstrably immature animal in a numerical cladistic analysis. Given the presence of plesiomorphic characters, we predict that it will be recovered in a position basal to *Diplodocus*. For this purpose, we used recent phylogenetic analyses as a starting point^11,12^ and analyzed the matrices using parsimony and Bayesian algorithms.

#### Institutional Abbreviations

AMNH, American Museum of Natural History, New York, NY; BYU, Brigham Young University, Provo, UT; CM, Carnegie Museum of Natural History, Pittsburgh, PA; CMC, Cincinnati Museum Center, Cincinnati, OH; HMNS, Houston Museum of Natural Science, Houston, TX; HMN, Humbolt Museum für Naturkunde, Berlin, Germany; MOR, Museum of the Rockies, Bozeman, MT; SMA, Sauriermuseum Aathal, Aathal, Switzerland; SMM, Science Museum of Minnesota, Saint Paul, MN; USNM, United States National Museum, Washington D.C.

## Results

### Description and comparisons

#### Skull

Most of the bones are identifiable, but portions of the maxillae, jugals, nasals, and lacrimals are damaged or missing.

The cranium of CMC VP14128 has diagnostic features of Diplodocidae including: a long prefrontal, paroccipital process with a rounded ventrolateral end, external nares that are retracted and dorsally-facing, a tooth row that does not extend the full length of the maxilla and dentary, low coronoid eminence, and absence of a squamosal-quadratojugal contact. Additionally, the morphology of the basal tubera (robust, triangular, and protruding posteroventrally from the basicranium), the presence of subnarial and maxillary foramina, and the largely peg-like teeth, while not exclusive to Diplodocidae, such morphologies and their combination are commonly observed in diplodocines.

To narrow down the taxonomic identity of CMC VP14128, we compared it with the four Morrison Formation genera of Diplodocinae: *Barosaurus*, *Diplodocus*, *Galeamopus*, and *Kaatedocus*. *Diplodocus* is known from eight complete skulls (CM 11255, CM 3452, CM 11161, MOR 7029, SMM P84.15.3, USNM 2672, USNM 2763), *Kaatedocus* from three skull (AMNH 7530 SMA 0004, SMA D-16/3^12,17^), and *Galeamopus* is reported from at least three partial skulls (AMNH 969, SMA 0011, and HMNS 175^12,27^). Fragmentary cranial remains of *Barosaurus* are reported from the Howe Quarry^12^ and recently have been recovered from the Aaron Scott Site in the San Raphael Swell of Utah (CMC VP15544). A tooth row not restricted to the anteriormost portion of the skull is present in CMC VP14128, *Kaatedocus* (SMA 0004), and *Galeamopus* (SMA 0011). The premaxillae of the newest and smallest specimen, CMC VP14128, express the Massopoda condition of four teeth - seen in *Diplodocus* and *Kaatedocus*. In contrast, the reconstructed skull of the *Galeamopus pabsti* holotype has five^27^. The exact morphology of the prefrontal in CMC VP14128 is difficult to determine due to taphonomic distortion, but it appears to exhibit the typical diplodocid posterior hook^27^. The anterior portion of the antorbital fenestra seems to be dorsally situated to the preantorbital fossa as seen in CM 11255, *Galeamopus*^12,27^ and possibly in *Kaatedocus*^17^, although the damaged margin in CMC VP14128 makes this observation tentative. The posterior margin of the postorbital is gracile and more forked – as in *Diplodocus* and *Kaatedocus*, in contrast to less forked in *Galeamopus*^12,27^; whereas the dorsomedial process is long and tapered as in *Diplodocus* and *Kaatedocus*^3,4,17^, but not *Galeamopus*^27^. The squamosal in CMC VP14128 has a tapered and long anterior process approaching the quadrate, as in *Diplodocus* and *Galeamopus*, and not *Kaatedocus* – an autapomorphy of this genus^17^. Due to taphonomic damage and distortion, the morphology of the sagittal nuchal crest cannot be accurately discerned in CMC VP14128 (distinct and narrow vs. wide^11,12^). In contrast, a distinct crest is found in both *Kaatedocus* and *Galeamopus hayi*^12,27^, and CMC VP14129 does exhibit this feature (see Materials and Methods). Therefore the weight of evidence indicates a referral to *Diplodocus* over the other taxa.

#### Mandible

The relative proportions of the dentary and surangular are very similar to the lower jaw of the larger, and slightly more mature *Diplodocus* (CM 11255^3^). Assuming similar proportions of mandibular bones in a mature *Diplodocus* to CMC VP14128, the skull would have been disproportionally stretched (see Material & Methods). Instead, CMC VP14128 indicates that in early ontogeny, the bones of the lower jaw were not directly proportionate to those of an adult (e.g. dentary to total jaw length). Regarding the dentary, the dorsoventral thickness post symphysis is more uniform as in *Diplodocus* and *Kaatedocus*^3,12^, opposed to strongly tapered as in *Galeamopus*^27^. One of the most interesting features of the lower jaw is the tooth row (Fig. 1B,C). As expressed in the upper jaw, the lower tooth row extends more posteriorly than seen in more mature specimens. In the right dentary of CMC VP14128, the tooth row (which is posteriorly obscured and damaged) is located along the anterior most ~1.5 cm. The left tooth row, however, clearly spans ~6.5 cm of the dentary; approximately 46% the length of the dentary compared to 22% in adults. Comparable to the adult condition, CM 11255 was hypothesized^3^ to have had 10–11 dentary teeth, whereas CMC VP14128 possesses 13. This dental variation may represent intraspecific variation^28^, considering that the dentary formula of CMC VP 14128 is the same as in immature camarasauromorphs^28^ and that ontogenetic dental formula reduction is documented in other dinosaurs^29,30^.

#### Dentition

The dental formula of CMC VP14128 is 4.8/13; the dental formula of the more mature CM 11255 has a formula of 4.8–9/10–11 dentary teeth, which is comparable to the adult condition of CM 11161 that has a formula of 4.9/11–12^31^.

**Figure 3:**
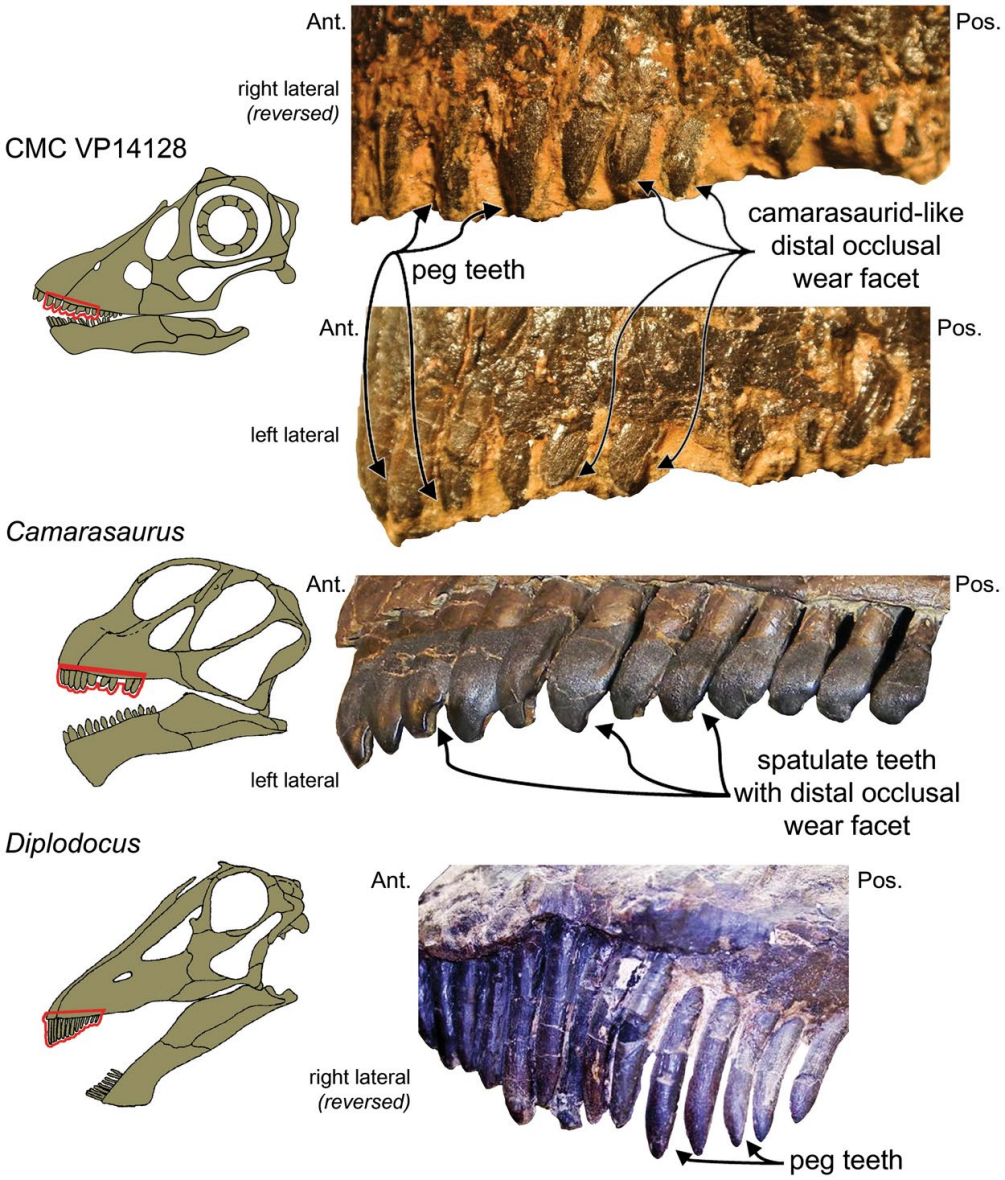
The dental morphotypes in CMC VP14128. Pre- and maxillary teeth of CMC VP14128 in right and left lateral. Drawing by K. Scannella. Red outlines highlight the zoomed in views on the right. Note the combination of diplodocid peg and camarasaurid spatulate tooth forms. *Camarasaurus* sp. with the spatulate tooth form (SMA 0002). *Diplodocus longus* with the peg tooth form (USNM 2672). *Camarasaurus* and *Diplodocus* skull modified from McIntosh^70^. Skulls not to scale.

The premaxillary teeth of CMC VP14128 exhibit the typical diplodocine condition: long, slightly inclined, pointed, and narrow-crowned – the so-called peg-like condition. However, from the second maxillary tooth posteriorly, the teeth are apicobasally short, with mesiodistally wide and more labial convex crowns. Several teeth have a *Camarasaurus*-like distal occlusal wear facet (Fig. 3). This relatively basal tooth morphology is consistent with the overall basal-expression form of the cranium.

### Maturational state of CMC VP 14128

CMC VP14128 establishes the immature condition for many features of the skull, jaws, dentition, and anterior cervical vertebrae far beyond what has been previously known. We summarize those changes here, and make note of immature features that correspond to the plesiomorphic character states of Diplodocoidea (for a greater discussion on the possibility of ontogenetic recapitulation, please consult the Supplementary Information).

#### Size

The estimated skull length of CMC VP14128 is 24.29 cm, which is ~40% the length of the largest adult *Diplodocus* skull (USNM 2673, ~60 cm). Cranial size differences observed between CMC VP14128 and adult skulls attest to changing body size through ontogeny (Fig. 2). The cranial size difference between CMC VP14128 and CM 11255 – ~5 cm – seems minor, yet the understanding of sauropodomorph paleobiology is dependent on their relative scale. Understanding minor skeletal nuances can have vast ontogenetic repercussions. A mere 25 cm difference in femoral length separates a 6 m 6-year-old from a 27 m 24-year-old *Diplodocus* (5).

While we do not have complete *Diplodocus* specimens, we have composites and referable material enabling us to draw some conclusions about adult proportions. Specifically, here we use *D*. *carnegii* CM 84 which is a composite, but represents the informal standard for the genus^32^. Nevertheless, using a ratio from CM 84 assumes isometric growth – contrary to the ontogenetic record of Dinosauria – therefore we should view the resulting estimates as nothing more than generalized proportions.

Using this adult cranial:body length ratio predicts a maximum body length of 9 m for CMC VP14128 and 10.9 m for CM 11255, a difference that would be even greater with a more realistic allometric skull-body length ratio; yet even this isometric trend indicates a minimum difference of nearly 2 m in body length, expressed in ~5 cm of cranial length-difference. While we await more specimens to fill in these crucial ontogenetic intervals, assuming size covaries with age at this locality (see Discussion), we hypothesize that CMC VP14128 was within the recorded MDQ ages of two – six years of age^5^ and had a body length well under the isometrically calculated 9 m (Fig. 1).

#### Tooth count

CMC VP14128 has a high dentary tooth count – 13 – in contrast to the lower tooth count – 11 – seen in larger, presumably more mature specimens, such as CM 11161 (see description above). This variation in tooth count, while limited in sample size, may be indicating a trend of dentary tooth count reduction, which is seen in other immature dinosaurs^29,30,33^. Therefore, the high dentary tooth count of CMC VP14128 indicates its juvenile growth stage. Also, basal eusauropods tend to have a dentary tooth count higher than 11^34,35^.

#### Neurocentral synostosis

Three cervical vertebrae with neural arches are preserved with CMC VP14128. In two of the cervical vertebrae (labeled 2 and 3 in Fig. S1) the arches are completely separate from their centra. One vertebra (labeled 1 in Fig. S1) has a fused arch with sutural contacts that are seen on the anterior- and posterior-most margins. The corresponding sutures in a skeletally mature *Diplodocus* (such as CM 84) are completely closed.

#### Cervical centrum pneumatization

The cervical centra of CMC VP14128 are excavated by shallow, simple, and weakly divided fossae, typical for young animals, as compared to highly complex fossae and foramina with numerous accessory laminae in adults such as *D*. *carnegii* CM 84^5,13,36–38^.

#### Cervical rib histology

In the absence of chronologically-informative bones (such as sauropod dorsal ribs that provide an almost complete growth record^39–41^), a cervical rib of CMC VP14128 was sectioned to obtain an estimate of the relative maturity of the specimen based on patterns of remodeling – in like manner to the Histologic Ontogenetic Stage^42^. However, we must cautiously note that the origin and development of cervical ribs is still ongoing research (JRH and DCW in prep.). As cervical ribs incorporate a complex developmental relationship of metaplastic and osteogenic processes^43,44^, at this time we should only compare rates of secondary remodeling (Fig. S2).

Progressing through maturity in diplodocid cervical ribs, there are dramatic changes in tissue composition. In the smallest specimen (SMA 0009) the tissue is composed entirely of highly vascular primary tissue. Progressing to CMC VP14128 the tissue is composed of secondary reconstructions and primary tissue – features indicative of metaplasia^45^. Finally, within a sub-adult *Diplodocus* (MOR 592), the tissue consists of regular bony tissues - a core of Haversian bone, periosteally grading from secondary to primary osteons (see greater discussion in the Supplementary Material).

While the ontogenetic development of cervical ribs must be studied in further detail, this analysis supports the hypothesis that they develop via metaplasia from a collagenous to an osseous tissue^43,44^. Thus, the cervical rib of CMC VP14128, conforming to this developmental pathway, further supports our maturational interferences of immaturity for this specimen.

Systematic Paleontology

Saurischia Seeley 1887

Sauropodomorpha von Huene 1932

Sauropoda Marsh 1878

Diplodocoidea Marsh 1884

Flagellicaudata Harris and Dodson 2004

Diplodocidae Marsh 1884

cf. *Diplodocus* Marsh 1878.

#### Comparative description

The Morrison Formation preserves three sauropod clades: Diplodocoidea, Camarasauridae, and Brachiosauridae. The lack of only spatulate teeth, an inclined posterior portion of the premaxilla, projecting external naris, cervical ribs shorter than centrum, and rectangular not rhomboidal cervical vertebrae profiles in CMC VP14128 are more diplodocoid than macronarian morphologies. Although many of the diagnostic characters in the sauropod skull are proportionally or ontogenetically variable^46^ some of the morphologies within CMC VP14128 are different from those expressed in the adult. While a few traits could be outside of the typical adult expressions, we hypothesize that characters/conditions of CMC VP14128 will at least largely associate with a known genus (as previously demonstrated in *Europasaurus*^47^).

CMC VP14128 is referable to Diplodocidae based on the presence of: a long posterior process of the prefrontal, teeth that do not span the length of the maxilla and dentary, a low coronoid eminence on the mandible, lack of crown-to-crown occlusion, cervical rib length that is shorter than the corresponding centrum length, and external nares that are retracted and face dorsally. CMC VP14128 is not referable to *Apatosaurus* given the presence of a basipterygoid recess, a basipterygoid process that lacks an anteroventral flare, and enlarged cervical ribs that do not project below the centrum. Likewise, CMC VP14128 is not referable to *Barosaurus*, based on the presence of long postzygapophyses, anteroposteriorly narrow neural spines, and a strongly posteriorly-angled centrum cotyles. CMC VP14128 has its strongest affinities with the slender diplodocines, including *Diplodocus*, *Galeamopus*, and *Kaatedocus*; however, the distribution of shared features is inconsistent, obscuring its lower-level identity. However, based on the number of shared characteristics, CMC VP14128 is most referable to *Diplodocus* than either *Galeamopus* or *Kaatedocus*.

However, it must be stated that specimens previously assigned to *Diplodocus*, and how we phylogenetically recognize and identify this genus are being reexamined^12,32^. Some historically recognized *Diplodocus* specimens are now being referred to other genera – such as USNM 2673 possibly representing *Galeamopus*^12^ and even CM 11255 to *Barosaurus*^48^. Additionally, some Morrison Formation taxa have little to any known or described cranial material (*Barosaurus*, *Dystrophaeus*, *Haplocanthosaurus*, *Supersaurus*, *Suuwassea*), therefore there are several taxa we cannot adequately compare CMC VP14128 to or assess. Additionally, while Whitlock^11^ identified three cranial autapomorphies for *Diplodocus* (preantorbital fenestra with well-defined fossa, pterygoid medial to ectopterygoid on transverse palatal hook, teeth inclined anteriorly relative to axis of jaw) since no skulls to date are unquestionably associated with post-crania, Tschopp *et al*.^12^ questioned these characters. While the latest phylogenetic analysis of Diplodocidae would advocated that no unambiguous diplodocinae cranial synapomorphies are recognized^12^, the exact taxonomic assignment of CMC VP14128 within Diplodocinae remains uncertain. With the current lack of no known diplodocinae synapomorphies^12^, one could taxonomically identify CMC VP14128 simply as diplodocinae indeterminate. However, given the predominance of similar morphologies between CMC VP14128 and *Diplodocus* sp. in comparison to the other Morrison Formation diplodocids, we tentatively opt to refer CMC VP14128 to cf. *Diplodocus*. While both identifications (diplodocinae indeterminate and cf. *Diplodocus*) are testable hypotheses, we currently believe that it is more fruitful and more constructive for future works to support/refute our identification of CMC VP14128 as cf. *Diplodocus* versus reanalyzing starting from a subfamily level identification.

### Phylogenetic analyses

#### Separate data sets

The cranial + postcranial parsimony and Bayesian analyses of the Whitlock^11^ data set recovered CMC VP14128 as the basalmost member of Dicraeosauridae, while the cranial only analysis recovered CMC VP14128 as the sister species of Dicraeosauridae, and *Dicraeosaurus* + *Amargasaurus*, respectively. The cranial + postcranial parsimony and Bayesian analyses of the Tschopp *et al*.^12^ data set recovered CMC VP14128, as a diplodocine more derived than *Diplodocus*, while the cranial only analysis recovered CMC VP14128 in a polytomy within Flagellicaudata.

#### Combined data set

**Figure 4:**
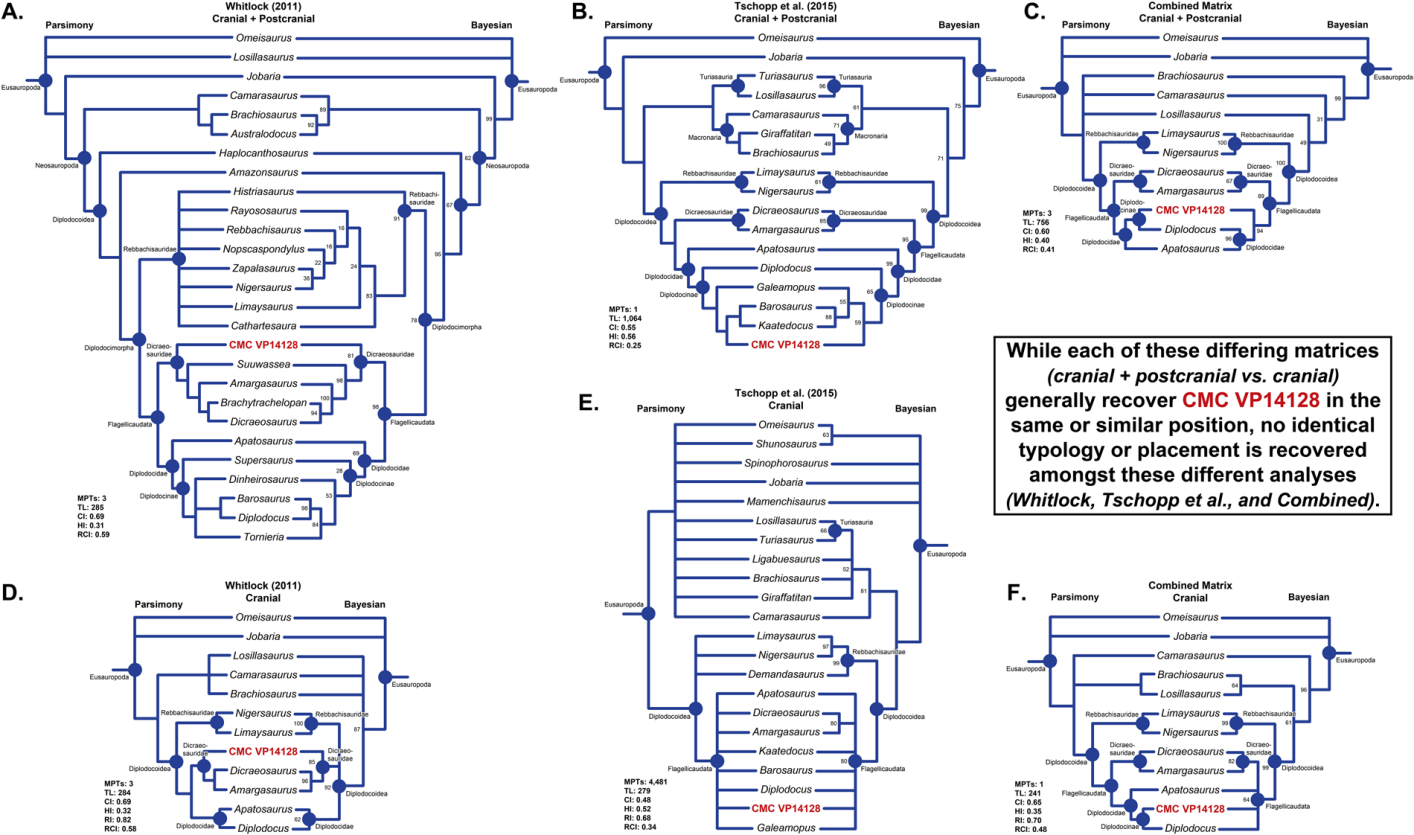
Dendrograms of parsimony (left column) and Bayesian (right column) phylogenetic analyses. (**A**–**C**) Consist of cranial and postcranial characters, while (**D**,**E**) consist of only cranial characters. (**A** and **D**) CMC VP14128 coded into the matrix of Whitlock^11^. (**B** and **E**) CMC VP14128 coded into the matrix of Tschopp *et al*.^12^. (**C** and **F**) CMC VP14128 coded into a combined matrix.

In the combined cranial + postcranial parsimony and Bayesian typology, there is a degree of basal uncertainty (no united Macronaria), yet there is a structured and organized Diplodocoidea (Fig. 4). In the parsimony typology, CMC VP14128 is recovered as the sister species to *Diplodocus*, while in the Bayesian analysis, it is recovered as a derived member of Flagellicaudata and basal to Diplodocidae. For the combined cranial only analyses, the typologies are similar to that of the cranial + postcranial analyses. There are distinct Rebbachisauridae, Dicraeosauridae, and Diplodocidae branches, and CMC VP14128 is recovered as the sister taxon to *Diplodocus*. Apart from minor changes in posterior probability, the Bayesian topology is very similar to the earlier analysis^12^. It likewise recovers CMC VP14128 in a flagellicaudatan polytomy in which Dicraeosauridae forms one branch. Yet we would caution that while these two analyses superficially produce similar results, the low support for groupings indicates that these relations are not definitive.

The minutia between the parsimony and Bayesian analyses do vary, but in general the encompassing skeletal analyses do little to elucidate the taxonomic identify of CMC VP14128. In the analysis of Whitlock^11^, CMC VP14128 is recovered in the same dicraeosaurid position, while in the analysis of Tschopp *et al*.^12^ taxonomy is slightly more refined as CMC VP14128 is recovered as a derived diplodocine versus a flagellicaudatan polytomy, and finally in the combined analysis, CMC VP14128 is still recovered as a diplodocoid. Certainly the encompassing analyses do reveal much more overall taxonomic resolution, but said resolution is largely irrespective to CMC VP14128. A cranial only approach could appear redundant or unnecessary, yet given the vastly differing phylogenetic placements between the matrices used herein, we would suggest that an elemental or regional styled analyses can be used to further check or verify specimens that are recovered in seemingly unusual or suspect positions.

## Discussion

### Phylogeny

Parsimony analyses of CMC VP14128 produced a conflict. One resulted in only a generic level association with *Diplodocus*, while the other advocated a unique basal position. If our cranial-only analyses represent true phylogenetic patterns, then relatively small morphological matrices (in terms of number of characters, percentage of each character region, and/or taxa) can be sensitive to inclusion of juvenile specimens^5^, as demonstrated by the more basal positioning of CMC VP14128. This is expected as even a few characters that change with ontogeny will have a large effect on the outcome of the analysis. In the larger^12^ and combined analyses, CMC VP14128 is recovered amongst other flagellicaudatans (including diplodocids), albeit in large polytomies, agreeing with our previous diagnosis. This suggests including additional characters can override the effect of a few characters that are ontogenetically biased, but as paleontological phylogenetic analyses are often based on small datasets, this bias can be significant.

In regard to the plesiomorphic characters in CMC VP14128, we theorize these might be evidence of recapitulation; i.e., the ephemeral presence of plesiomorphic characters that transition into their derived states by adulthood. Recapitulation has been documented in other basal^49,50^ and derived^47,51–53^ immature sauropods. Morphologies in CMC VP14128 – such as the posterior tooth row, lower jaw proportions, and rounded snout – could be recapitulatory in origin (see Supplementary Material); yet given the relationship between cranial attributes and feeding strategy, these morphologies could also derive from ontogenetic ecomorphological relationships (see below). More specimens and analyses are need to substantiate either possibility, and we note that these hypotheses are not mutually exclusive.

Despite the presence in CMC VP14128 of phylogenetically plesiomorphic features that are not seen in adult *Diplodocus*, this does not mean that the entire skeleton of CMC VP14128 is plesiomorphic, (or that it is a misidentified basal taxon), since the specimen does have characters of *Diplodocus*. Instead, this combination of characters is evidence that 1: not all parts of the skeleton develop at the same rate, and 2: regardless of rate, ontogenetic skeletal changes followed the phylogenetic progression from plesiomorphic to derived states. Recognition of this phenomenon strongly cautions against diagnosing new taxa based on small statured specimens displaying a combination of basal and derived characters, which might instead be evidence of immaturity. Interestingly, similar findings were reported by Tschopp *et al*.^12^ regarding “*Elosaurus*” *parvus* (CM 566). While originally thought to be a valid taxon, “*Elosaurus*” is now regarded as an immature *Brontosaurus parvus*^12^ (however, while this analysis believes this maturational inference is highly likely, we cautiously note that this hypothesis has not been histologically tested). Including CM 566 into a specimen-level analysis, Tschopp *et al*.^12^ recovered the specimen not only within the species *B*. *parvus*, but also as the basalmost specimen. We would agree with Tschopp *et al*.^12^ that more studies are needed to address this issue, and this further highlights the multifaceted relationship between ontogenetically variable characters and taxonomic recovery.

### Ecomorphology and behavior implications

The unique cranial and dental characters seen in the immature CMC VP14128 suggests resource partitioning between juvenile and adult *Diplodocus*. Like the immature *Diplodocus* CM 11255^3^, CMC VP14128 has a mediolaterally narrow snout, in stark contrast to more mature individuals that express a wide and squared snout. These differences have been hypothesized to indicate dietary niche-partitioning through ontogeny^3^, and the narrow snout of CMC VP14128 is consistent with the hypothesis that immature diplodocids had a selective feeding strategy, while fully mature animals were ground-level browsers^3^. CMCVP14128 also has spatulate teeth and an extended tooth row. Spatulate teeth are more efficient for coarse vegetation and bulk feeding, and non-spatulate teeth are beneficial for softer foliage and browsing^54,55^. This combination of dental morphologies in CMC VP14128 may indicate that very immature diplodocids were feeding on a greater variety of plant materials, and orally processed them differently than their more mature counterparts.

However, previous studies show that nutrition selectivity is problematic in animals exceeding a certain size and that there is a selection pressure towards browsing for large herbivores^56,57^. Another recent analysis^58^ showed that narrow, short, and rounded macronarian snouts were more efficient for forested environments while broader and longer diplodocoid snouts suggest open, ground-level browsing. This could also be interpreted as indicators of different ecological environments, implying macronarians would have been adapted for more forested environments while diplodocids were more specialized for open environments. Therefore, the plesiomorphic macronarian-like characters seen in CMC VP14128 could also indicate that juvenile diplodocids lived in more forested environments than the adults that (restricted and protected by their size) were most likely browsing in more open habitats. Foraging in forests would have provided the juveniles with protective cover from predators, a danger that colossal adults would not share.

The skull and tooth morphology of *Diplodocus* suggests that these animals transitioned through distinct feeding roles over their lifespan. This inference is supported by a study of *Alligator mississippiensis* tooth change through ontogeny^59^. Dental changes coincided with changes in diet^59–61^, thus dental allometry could be attributed to dietary partitioning.

Though it is currently unknown which specific plant species Morrison Formation sauropods ate, based on δ^13^C relationships between modern plant equivalents and tooth enamel implies that *Diplodocus* may have fed on ferns and horsetail, while *Camarasaurus* was generalized, feeding on a wide range of foliage, including ferns, horsetails, conifers, and cycads^62^. An immature *Diplodocus* may have fended for itself (or possibly as part of an age-segregated herd^63^) and fed on differing foliage to gain more nutrients during critical development.

The ontogenetic change in dental morphologies observed within *Diplodocus* also gives evidence for the lack of parental care in sauropods (along with nest structures and histology^14,64^). If adults fed hatchlings - potentially the foliage they ate - then there would be no reason for the changes in tooth morphology. Evidence of precocial juvenile sauropods was also found in a recent histologic assessments of a very immature titanosaur. Furthermore, the extreme size differences between parent and hatchlings could have resulted in high infant mortalities due to trampling.

The differences in cranial morphology between CMC VP14128 and more mature *Diplodocus* specimens (such as CM 11255; Whitlock *et al*.^3^) highlights extreme cranial changes that occurred rapidly over a short increase in size. In addition to the narrow snout, the proportionally enlarged braincase and extremely large orbits are both infantile attributes observed in many other immature vertebrates. The more box-like cranial condition and the extended tooth row in CMC VP14128 are reminiscent of the co-occurring camarasaurid condition. If these characters represents recapitulation, we could hypothesize that dietary generalization is the more basal condition and increasing specialization the more derived.

### Possible dwarfism?

Instead of a juvenile, CMC VP14128 could represent an unknown Morrison Formation dwarf taxon. Two separate investigations of age determinant histology and morphology of the MDQ material produced different results^5,41^. The bone tissue types in the first analysis indicate average sized immature diplodocids with typically expressed tissues^5^, yet many of the specimens of the second analysis exhibit tissues indicating skeletal maturity^41^. In comparing histology, this is not simply the case of incorrect tissue interpretations (DCW and KW pers. obs.) – the varying tissue morphologies indicate a more complex story for the MDQ.

The regional geology does not support the presence of an island depositional system – an extensive marine unit with localized terrestrial deposits. Nevertheless, regional geography or environment could explain a possible size difference. In the case of stegosaur specimens from the northern extent of the Morrison Formation, larger overall size (*Stegosaurus*) appears correlated with arid environments, and smaller size with wetter climates (*Hesperosaurus*^65^). Based on the coastal setting adjacent to the Sundance Seaway, this trend in stegosaur size was hypothesized to be environmentally correlated^65^. A similar pattern might hold true for the MDQ diplodocids.

Other Morrison Formation localities in Montana record more typical sized specimens of *Apatosaurus* and *Diplodocus*^5,66^. This regional size difference, and size difference within the MDQ^41^, may represent regional variation. Such regional differences are present in a modern group of herbivores, cervids. The North American coastal White-tailed Deer (*Odocoileus virginianus*) have an average body mass 77 kg, while their interior counterparts have an average body mass in excess of 100 kg^67^. The smallest sub-species, the Key Deer (*Odocoileus virginianus clavium*) represents an example of island dwarfing has an average body mass of 34 kg^67^. Therefore, these size differences between localities may indicate variably-sized regional diplodocid populations. In spite of these findings CMC VP14128 is almost certainly not a regional dwarf because it lacks autapomorphies. The morphologies we assigned to ontogeny could hypothetically be used to erect autapomorphies, however we strongly caution that the onus is on such an analysis to demonstrate that no attributes are related to ontogenetic variation as proposed herein. Alternatively, opposed to the dwarf morphotype observed by Waskow *et al*.^41^, CMC VP14128 may represent the larger morph present at the site.

## Conclusions

**Figure 5:**
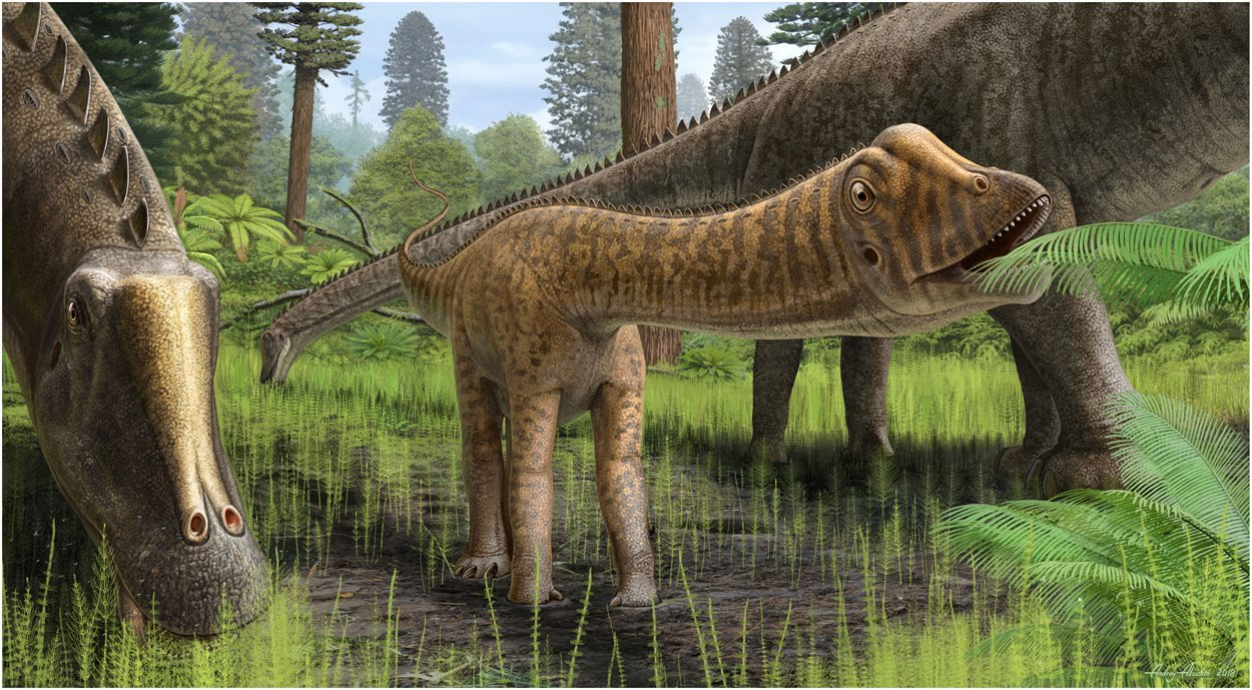
Life reconstruction of CMC VP14128. Note the cranial morphologies interpreted to denote differing feeding strategies: in CMC VP14128 the narrow snout with posteriorly elongated and morphologically varied tooth row for bulk feeding vs. the widened snout with anteriorly restricted peg-shaped teeth for ground-level browsing in adults. Also note the camouflaged ontogenetic color change suggesting young diplodocids may have sought forested refuge. Reconstruction by A. Atuchin.

Within Dinosauria, there are small bodied taxa that display basal and derived characters and occupy unusual basal phylogenetic positions. The validity and position of such taxa has been disputed^21–26,68,69^, and regarding sauropodomorph phylogeny, we would advocate that the combination of basal and derived characters and basal phylogenetic recovery should be recognized as an indicator of an immature ontogimorph – instead of a distinct taxon. In light of the current wealth of information pertaining to dinosaur ontogeny, we can no longer assume that all morphological differences correspond with phylogenetic distinctiveness. Accounting for ontogeny could prove as test for our phylogenies. Recognizing the ontogenetic age of immature specimens provides important insights into the life history of these animals. The immature *Diplodocus* specimen CMC VP14128 extends our understanding of the ontogeny of the genus and the evolution of diplodocids into new areas, where:

1. The combination of basal and derived characters in the juvenile is broadly congruent with the phylogenetic transition from eusauropods to diplodocoids.
2. The plesiomorphic tooth morphology is retained in immature *Diplodocus* and lost with maturity, and we predict this growth pattern will be seen in all other diplodocoids.
3. As first proposed by Whitlock *et al*.^3^, tooth and skull morphology indicate that during growth *Diplodocus* inhabited different trophic levels/niches, where juveniles were generalists (i.e., browsers; Fig. 5) and more mature individuals were specialists (i.e., ground-level browsing), a pattern that we predict is ancestral for Diplodocoidea.

## Electronic supplementary material

Supplementary Information

Supplementary Information S1

Supplementary Information S2

Supplementary Information S3
