# Supplementary material for: The Smallest Diplodocid Skull Reveals Cranial Ontogeny and Growth-Related Dietary Changes in the Largest Dinosaurs
Source: Sci Rep. 2018 Oct 11;8:14341. doi: 10.1038/s41598-018-32620-x (PMC6181913; doi:10.1038/s41598-018-32620-x)
Supplement: Supplementary file 1 — Supplementary Information [file 41598_2018_32620_MOESM1_ESM.docx]

The Smallest Diplodocid Skull Reveals Cranial Ontogeny and Growth-Related Dietary Changes in the Largest Dinosaurs

D. Cary Woodruff^1,2,3^*, Thomas D. Carr^4^, Glenn W. Storrs^5^, Katja Waskow^6^, John B. Scannella^7^, Klara K. Nordén^8^, John P. Wilson^9^

Supplemental Information

**Phylogenetic analysis**

We tested the taxonomic identity of CM VP14128 by including it in the phylogenetic analyses of Whitlock^11^ and Tschopp *et al*.^12^. Additionally we used modified matrices from Whitlock^11^ and Tschopp *et al*.^12^ consisting of only cranial characters. Finally, we combined these matrices (a cranial + postcranial and a cranial only matrix), as a larger matrix may circumvent problems with ontogenetically changeable characters.

**Cranial + postcranial characters**

We included CM VP14128 in the data matrix that combined the matrices of Whitlock^11^ and Tschopp *et al*.^12^ that included two outgroup taxa, 10 ingroup taxa, and 540 characters; following that analysis, *Omeisaurus* and *Jobaria* were designated as outgroup taxa. We ran the matrix in PAUP with the characters unordered under a branch and bound search, a furthest addition sequence, zero length branches were collapsed, and the ‘MulTrees’ option was in effect.

For the “Combined Matrix Cranial + Postcranial” matrix we obtained three most parsimonious trees, each with a tree length of 756 steps, with a CI (excluding uninformative characters) of 0.60, an HI (excluding uninformative characters) of 0.40, and an RCI of 0.41. We obtained a strict consensus tree that showed a resolved Diplodocoidea and an unresolved polytomy of basal neosauropods. The position of CM VP14128 is resolved as the sister species of *Diplodocus*, a relationship supported by the common possession of the following unambiguously optimized characters: tooth crown aligned along jaw axis such that the crowns do not overlap, cervical vertebrae possess a longitudinal sulcus on their ventral surface, a lamina divides the pleurocoel on the cervical vertebrae, paired pneumatic fossae are present on the ventral surface of the anterior cervicals, posterior cervical neural arches lack an accessory spinal lamina , and the anterior and mid-caudal centra possess a ventral longitudinal hollow. The characters in code form that support the position of CM VP14128: 68 (0 to 1), 79 (0 to 1), 82 (0 to 1), 89 (0 to 1), 98 (1 to 0), 144 (0 to 1).

We included CM VP14128 in the data matrix of Tschopp *et al*.^12^, and we followed their procedure in ordering 23 characters (see Tschopp *et al*.^12^) and in designating *Shunosaurus* as the outgroup taxon. The matrix includes 22 taxa and 477 characters. We ran the matrix in PAUP under a branch and bound search, a furthest addition sequence, zero length branches were collapsed, and the ‘MulTrees’ option was in effect.

For this matrix we obtained a single most parsimonious tree that has a CI of 0.55, and HI of 0.56, an RCI of 0.25, and a tree length of 1,064 steps. The position of CM VP14128 is resolved as the sister species of *Kaatedocus* + *Barosaurus*, which is supported by five unambiguously resolved characters: 24 (0 to 1), 32 (1 to 0), 74 (0 to 1), 87 (1 to 0), 154 (1 to 0).

Finally, we included CM VP14128 in the data matrix “Whitlock 2011 Cranial + Postcranial” [Cary: insert how you want to identify this matrix instead of the file name I’ve used], which includes 27 taxa and 192 characters, *Omeisaurus* and *Jobaria* were designated as outgroups and we followed the same procedures as for the other analyses.

We obtained three most parsimonious trees, each with a CI of 0.69, HI of 0.31, and and RCI of 0.59, and a length of 285 steps. The position of CM VP14128 is resolved as the sister species of the (*Suuwassea* (*Amargasaurus* (*Brachytrachelopan* + *Dicraesaurus*))) clade. The position of CM VP14128 is supported by five unambiguously optimized characters: 24 (1 to 0), 37 (0 to 1), 40 (0 to 1), 45 (0 to 1), 60 (0 to 1).

**Cranial only characters**

Using one published parsimony analysis dataset^11^ recovered a topology nearly identical to that originally recovered, where CMC VP14128 is recovered as the basalmost member of Dicraeosauridae (Fig. 4). While the Bayesian analysis of this matrix recovered relatively high support for CMC VP14128 as a sister to *Amargasaurus* and *Dicraeosaurus* (Dicraeosauridae), while the diplodocids *Diplodocus* and *Apatosaurus* fall out as a sister to this branch.

Next, we coded CMC VP14128 into a second diplodocoid matrix^12^, but with an increased number of characters (127 vs. 76) and taxa/specimens (81 vs. 26). Theoretically, such an increased amount of information should result in greater phylogenetic resolution. Both this analysis and the Bayesian analysis recovered a similar topology in that all members of Flagellicaudata, including CMC VP14128, were recovered together in a singular, unresolved polytomy (Fig. 4).

The combining of matrices entails bringing together the taxa and characters to create a larger, more encompassing matrix^71^. Accounting for shared characters, our combined matrix consisted of 153 cranial characters. Regarding taxa, we only used those that both analyses shared. While maximum parsimony is currently the most popular method used for morphological datasets, Bayesian methods have alternatively been argued to show a higher accuracy^72–74^. We hence analyzed the data with both methods.

For the parsimony analysis we coded CMC VP14128 and incorporated it into Whitlock’s^11^ phylogenetic analysis of Diplodocoidea. Following that work we designated *Omeisaurus* and *Jobaria* as outgroups and excluded the redundant taxa *Rayosaurus* and *Nopscaspondylus*. The data matrix was constructed in MacClade^75^ and executed in PAUP v.4.0^76^. We ran a branch-and-bound search with the characters unordered and unweighted under ACCTRAN optimization.

In the resulting basal dicraeosaurid location, the unambiguously optimized characters that support this position includes: presence of frontal contribution to the supratemporal fenestra; prominent and ventrally directed process from the squamosal extending from its posteroventral margin; postparietal foramen present; a narrow, sharp, and distinct sagittal crest of the supraoccipital; and subtriangular cross section of the dentary, tapering sharply towards its ventral extreme.

Next, we coded CMC VP14128 into the matrix of Tschopp *et al*.^12^. Like Whitlock^11^, Tshopp et al.^12^ also examined the phylogenetic relationship of diplodocids, but with an increased number of characters (127 vs. 76) and taxa/specimens (81 vs. 26). Theoretically, such an increased amount of information (if phylogenetically informative) – here in the form of characters and comparative individuals – should result in greater phylogenetic resolution. Bayesian analyses were run in MrBayes v3.2^77^. As the dataset did not include constant characters we applied the Mkv model^78^. For each data set we ran the analysis for 2 million generations (four chains, two runs) with a sampling frequency of 1,000. Priors were kept to standard settings. We tested both a model with equal rates of character change, and a model with variable rates (gamma distribution). The support for each model was then evaluated using the Bayes factor, with a value over 10 considered as strong support for either model^79^. We checked log-likelihood scores to ensure the analysis had reached a stable phase (visually examining plots). Convergence between runs was judged when ESS (Estimated Sample Size) was greater than 200, PSRF (Potential Scale Reduction Factor) was 1, and average standard deviation of split frequencies was less than 0.1. The trees generated during the first 10,000 generations were discarded as burn in for the compilation of the majority-rule consensus tree.

**Vertebrae**

The post-cranial remains of CMC VP14128 comprise vertebrae consisting of an incomplete proatlas and at least four anterior cervicals (Fig. S1 and Fig. S2). Taphonomically, it is interesting to note that although the skull, proatlas, and anterior cervicals are preserved, the median bones (atlas and axis vertebrae) are missing. The vertebrae bear morphologies diagnostic of the Morrison Formation members of Diplodocidae, and are clearly dissimilar from the sympatric macronarians (*Brachiosaurus* – better cervicals known from the African *Giraffatitan*, HMN SII^80^), *Camarasaurus* [such as BYU 9047^81^]). While the vertebrae of CMC VP14128 are anteroposteriorly long (as also in *Giraffatitan*), macronarian cervicals have a strong ventral curvature to the centra with angled condyles, cotyles, and neural arches that result in largely rhomboidal lateral profiles^80,81^ – inconsistent with the rectangular diplodocid condition^12,17,27,82–84^.

The centra are accamerate to slightly procamerate, and the pneumatic fossae are simple and weakly divided by an accessory lamina (conditions observed in immature diplodocids^5^). The cervical centrum length is approximately two times longer than cotyle width. The ventral surfaces of the centra exhibit a slight lateral concavity, and all lack an anteroposterior ventral keel (as seen in *Apatosaurus*^83,84^). Unlike the diplodocinae *Barosaurus* (AMNH 6341^85^), in lateral view the cotyle is strongly angled dorsally to the centrum. In the one complete neural arch, the prezygapophyses do not project anterior to the condyle, yet the postzygapophyses are anteroposterioly long (unlike *Barosaurus*) and project posteriorly well past the cotyle, and display prominent epipophyses (*Diplodocus* conditions^82^). The diapophysis is strongly posteriorly oriented, while the angle between the prezygapophyses and neural spine is V-shaped and very steep (*Diplodocus* conditions^82^). The neural spines are not bifurcated (a spinal feature ubiquitous in all diplodocids); although this lack of bifurcation is attributable to the specimen’s ontogenetic status^5,86,87^).

All cervical ribs in articulation or associated are shorter than their respective centra (a condition expressed in Diplodocidae; although rare, cervical ribs longer than the centra have infrequently been observed in some immature diplodocids(^87^; pers. obs. DCW). The cervical ribs exhibit an overall gracile morphology. They do not project strongly ventrally from the centrum, nor do they exhibit a pronounced ventrolateral process or a robust anterior process (conditions typically observed in *Apatosaurus*^83,84^. The shaft of each cervical rib exhibits a slight ventral curvature, maintains a general uniform width, and tapers gradually (a condition more typical of the Diplodocinae than Apatosaurinae conditions). Likewise, the minor mediolateral width of the capitulum (nearly 1.5 times smaller than the length of the tuberculum) produces a dorsoventrally narrow and oval-shaped costotransverse ansa (more analogous to *Diplodocus* *carnegii*^82^ than *Apatosaurus* *ajax*^84^).

Of the represented cervical vertebrae, three are preserved in good condition. In two of these the neural arch is completely unfused, and in only one of these is there a fragmentary portion of the arch. The best preserved cervical has a fused arch with sutural contacts only visible on the anterior- and posterior-most margins (Fig. S1). While developmental trends in vertebral synostosis do appear within sauropods^48,88^, numerous dinosaur specimens – including CMC VP14128 – exhibit variability, and therefore indicate this feature’s plasticity and ambiguity regarding the use of fusion alone as an ontogenetic indicator.

**Cervical rib histology**

As demonstrated within sauropod dorsal ribs^39^ and cervical ribs^44^, serial location is critical for accurate comparisons. The microstructure from the anterior and posterior regions greatly differs as the cervical rib documents the transition from osteological to tendinous tissues^44^. All samples were taken as near mid-shaft as possible. Cross-sectional shape differences likely reflect some of this variation in sample locations (the MOR 592 sample – Fig. S3 C – is the most anterior of the set), yet variation within the sampled region should still produce comparable sections.

In a cervical rib of the smallest diplodocid postcranium known to date (the taxonomically debated SMA 0009), the tissue is composed entirely of highly vascular primary tissue (Fig. S3 A). Amongst the numerous collagen fibrils and fibroblast lacunae are Haversian canals with resorptive edges. Within CMC VP14128 we see interstitial material and structures initially resembling secondary osteons. However, upon closer inspection these structures lack osteocyte lacunae and canaliculi, and they do not possess a true cement line. Demarcated borders exist, but range from sinuous to not fully encompassing (Fig. S3 B). Instead we identify these features as secondary reconstructions^45^. In examining the interstitial material, it is a very fibrous or woven-looking tissue. Like the secondary reconstructions, this tissue is devoid of lacunae and is entirely composed of irregularly sized and spaced hypermineralized collagen fibrils; as such we identify this as primary tissue^45^. As secondary reconstructions and primary tissue are indicative of metaplasia, we suggest that the cervical rib of CMC VP14128 is largely – if not entirely – composed of metaplastic tissues. Finally, in a 12 year old *Diplodocus*^86^, we see the core of the cervical rib is composed of Haversian bone, and periosteally grading from multiple to single generational secondary osteons, with primary osteons constituting the outermost tissue (Fig. S3).

**Phylogenetic recapitulation during ontogeny**

As discussed in the main article, CMC VP14128 possesses phylogenetically plesiomorphic features that are not seen in adult *Diplodocus*. While these traits could derive from ecomorphological relationships, they could alternatively represent phylogenetic recapitulation. Recapitulation, as used here, refers to the presence of plesiomorphic character states that are seen in early stages of ontogeny that are seen to be replaced later in growth by their derived character states. Therefore, two criteria are required to pass the test to qualify as recapitulated characters, namely 1) the equivalents of the phylogenetic characters are seen in growth, and 2) the derived state must be preceded by the plesiomorphic state.

For example, CMC VP14128 possesses several features that are equivalent to plesiomorphic character states relative to Diplodocoidea that are replaced in growth by the descendant character states; these include: narrow and short snout (wide and long in adults), dorsoventrally tall maxilla and premaxilla (long and low in adults), small antorbital fenestra (large in adults), mesiodistally long tooth rows (mesially limited in adults), spatulate maxillary teeth (peg-like in adults), and simply pneumatized cervical centra (complexly pneumatic in adults). Since the juvenile features seen in CMC VP14128 correspond to the non-diplodocoid condition, as is seen in sauropod phylogeny, then this would support the hypothesis of recapitulation in the post-hatching growth of *Diplodocus*.

Additionally, the change in the ecomorphotype of the skull from the juvenile to adult condition is consistent with the phylogenetic progression, which is the predicted pattern if recapitulation is at work. Otherwise, a matching pattern between ontogeny and phylogeny would not be seen. Therefore, the phylogenetic transition is incorporated into the developmental pathway, and, as a result, constrains the growth pattern. As a result of this phenomenon, the juveniles and adults are ecomorphologically separated from each other and so they do not compete for the same resources.

**Literature Cited**

5. Woodruff, D. C., Fowler, D. W. & Horner, J. R. A new multi-faceted framework for deciphering diplodocid ontogeny. *Palaeontol. Electron.* **20.3.43A,** 1–53 (2017).

11. Whitlock, J. A. A phylogenetic analysis of Diplodocoidea (Saurischia: Sauropoda). *Zool. J. Linn. Soc.* **161,** 872–915 (2011).

12. Tschopp, E., Mateus, O. & Benson, R. B. J. A specimen-level phylogenetic analysis and taxonomic revision of Diplodocidae (Dinosauria, Sauropoda). *PeerJ* **3,** e857 (2015).

17. Tschopp, E. & Mateus, O. The skull and neck of a new flagellicaudatan sauropod from the Morrison Formation and its implication for the evolution and ontogeny of diplodocid dinosaurs. *J. Syst. Palaeontol.* **11,** 853–888 (2013).

27. Tschopp, E. & Mateus, O. Osteology of *Galeamopus pabsti* sp. nov. (Sauropoda: Diplodocidae), with implications for neurocentral closure timing, and the cervico-dorsal transition in diplodocids. *PeerJ* **5,** e3179 (2017).

39. Waskow, K. & Sander, P. M. Growth record and histological variation in the dorsal ribs of *Camarasaurus* sp. (Sauropoda). *J. Vertebr. Paleontol.* **34,** 852–869 (2014).

44. Klein, N., Christian, A. & Sander, P. M. Histology shows that elongated neck ribs in sauropod dinosaurs are ossified tendons. *Biol. Lett.* **8,** 1032–1035 (2012).

45. Horner, J. R., Woodward, H. N. & Bailleul, A. M. Mineralized tissues in dinosaurs interpreted as having formed through metaplasia: A preliminary evaluation. *Comptes Rendus - Palevol* **15,** 183–203 (2016).

71. Brusatte, S. L. & Carr, T. D. The phylogeny and evolutionary history of tyrannosaurid dinosaurs. *Sci. Rep.* **6,** 1–8 (2016).

72. Wright, A. M. & Hillis, D. M. Bayesian Analysis Using a Simple Likelihood Model Outperforms Parsimony for Estimation of Phylogeny from Discrete Morphological Data. *PLoS One* **9,** (2014).

73. O’Reilly, J. E. *et al.* Bayesian methods outperform parsimony but at the expense of precision in the estimation of phylogeny from discrete morphological data. *Biol. Lett.* **12,** 20160081 (2016).

74. Puttick, M. N. *et al.* Uncertain-tree: discriminating among competing approaches to the phylogenetic analysis of phenotype data. *Proc. R. Soc. B Biol. Sci.* **284,** 20162290 (2017).

75. Mindell, P. MacClade: Analysis of Phylogeny and Character Evolution. *Auk* **111,** 1035–1036 (1994).

76. Swofford, D. L. PAUP*: Phylogenetic Analysis Using Parsimony (*and Other Methods). *Sinauer Assoc. Sunderland, Massachusetts.* 1–142 (2002).

77. Ronquist, F. *et al.* Mrbayes 3.2: Efficient bayesian phylogenetic inference and model choice across a large model space. *Syst. Biol.* **61,** 539–542 (2012).

78. Lewis, P. O. A likelihood approach to estimating phylogeny from discrete morphological character data. *Syst. Biol.* **50,** 913–925 (2001).

79. Kass, R. E. & Raftery, A. E. Bayes factors. *J. Am. Stat. Assoc.* **90,** 773–795 (1995).

80. Janensch, W. Die wirbelsäule von *Brachiosaurus* *brancai*. *Palaeontogr. (Supplement 7)* **3,** 27–93 (1950).

81. McIntosh, J. S., Miller, W. E., Stadtman, K. L. & Gillette, D. D. The Osteology of *Camarasaurus* *lewisi* (Jensen, 1988). *BYU Geol. Stud.* **41,** 73–115 (1996).

82. Hatcher. *Diplodocus* Marsh, its osteology, taxonomy, and probable habits, with a restoration of the skeleton. *Mem. Carnegie Museum* **1,** 1–64 (1901).

83. Gilmore, C. W. Osteology of *Apatosaurus*, with special reference to specimens in the Carnegie Museum. *Mem. Carnegie Museum* **11,** 175–300 (1936).

84. Upchurch, P., Tomida, Y. & Barrett, P. M. A new specimen of *Apatosaurus* *ajax* (Sauropoda: Diplodocidae) from the Morrison Formation (Upper Jurassic) of Wyoming, USA. *Natl. Sci. Museum Monogr.* **26,** 1–108 (2004).

85. McIntosh, J. S. in *Thunder-lizards: The Sauropodomorph Dinosaurs* 38–77 (2005).

86. Woodruff, D. C. & Fowler, D. W. Ontogenetic influence on neural spine bifurcation diplodocoidea (dinosauria: Sauropoda): A critical phylogenetic character. *J. Morphol.* **273,** 754–764 (2012).

87. Mannion, P. D., Upchurch, P., Mateus, O., Barnes, R. N. & Jonese, M. E. H. New information on the anatomy and systematic position of *Dinheirosaurus* *lourinhanensis* (Sauropoda: Diplodocoidea) from the Late Jurassic of Portugal, with a review of European diplodocoids. *Journal of Systematic Palaeontology* **10,** 521–551 (2012).

88. Hanik, G. M., Lamanna, M. C. & Whitlock, J. A. A Juvenile Specimen of *Barosaurus* Marsh, 1890 (Sauropoda: Diplodocidae) from the Upper Jurassic Morrison Formation of Dinosaur National Monument, Utah, USA. *Ann. Carnegie Museum* **84,** 253–263 (2017).

**Figure Captions**

Supp. Info. Figure 1: Post cranial remains of CMC VP14128. For both vertebrae A (right lateral), B (left lateral), C (anterior), D (dorsal), E (ventral), F (posterior). Note the numbering of the cervical vertebrae (1-2) does not correspond serially, merely a numerical way to denote each element.

Supp. Info. Figure 2: Post cranial remains of CMC VP14128. A (right lateral), B (left lateral), C (anterior), D (dorsal), E (ventral), F (posterior). Note the numbering of the cervical vertebrae (3-4) does not correspond serially, merely a numerical way to denote each element. The centrum of cervical 4, proatlas, and cervical ribs only shown in a single plane.

Supp. Info. Figure 3: Histology of diplodocid cervical ribs. Blue boxes denote the magnified areas to the right of each specimen. A. SMA 0009; B. CMC VP14128; C. MOR 592. H.C. w/ Scal. Ed. = Haversian canals with scalloped edges; Col. Fib. = collagen fibrils; 2^nd^ Rec. = secondary reconstructions; Pri. Tis. = primary tissue; Pri. Ost. = primary osteons; 2^nd^ Ost. = secondary osteons. Note the hypothesized ontogenetic development from a metaplastic to osseous tissue. Specimens not to scale.

Supp. Info. Figure 4: A. Linear regression of diplodocid lower jaws to skull length. B. Linear regression of diplodocid skull to estimated body length.

Supp. Info. Figure 5: CM VP14128 (top) with overlay grid and landmark locations (red circles). Adult *Diplodocus* (bottom; redrawn from *17*) with transformed grid and landmark locations (red circles). Drawings by K. Scannella.

Supp. Info. Figure 6: The second specimen found in the field jacket with CMC VP14128, CMC VP14129. Note that CMC VP14129 was found on the opposing side of the jacket, and no other material was found with this specimen. A (dorsal), B (right lateral), C (left lateral), D (ventral), E (anterior), F (posterior).

Measurement Figure 1: Select measurements for Part 1 (consult Fig. 1 for numbering scheme) of CMC VP14128. Colors for each measurement correspond to the lines in the corresponding image.

Measurement Figure 2: Select measurements for Parts 2 and 3 (consult Fig. 1 for numbering scheme) of CMC VP14128. Colors for each measurement correspond to the lines in the corresponding image.

Measurement Figure 3: Select measurements for Part 4 (consult Fig. 1 for numbering scheme) of CMC VP14128. Colors for each measurement correspond to the lines in the corresponding image. Drawings by K. Scannella.

Character Matrices (*excel spreadsheets*)

Photogrammetric model 1: (*3D pdf*)

Photogrammetric model 2: (*3D pdf*)


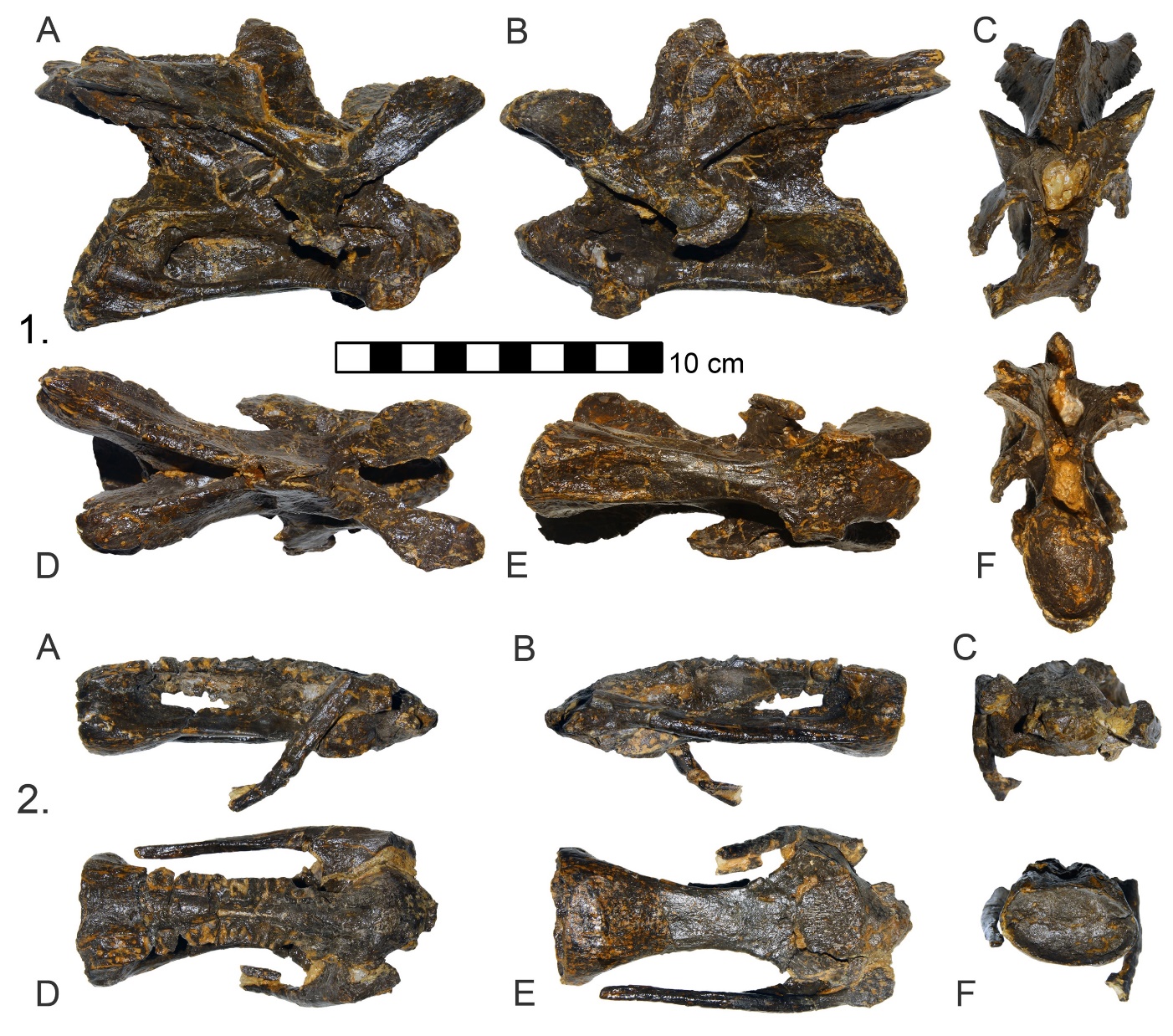


S.I. Fig. 1


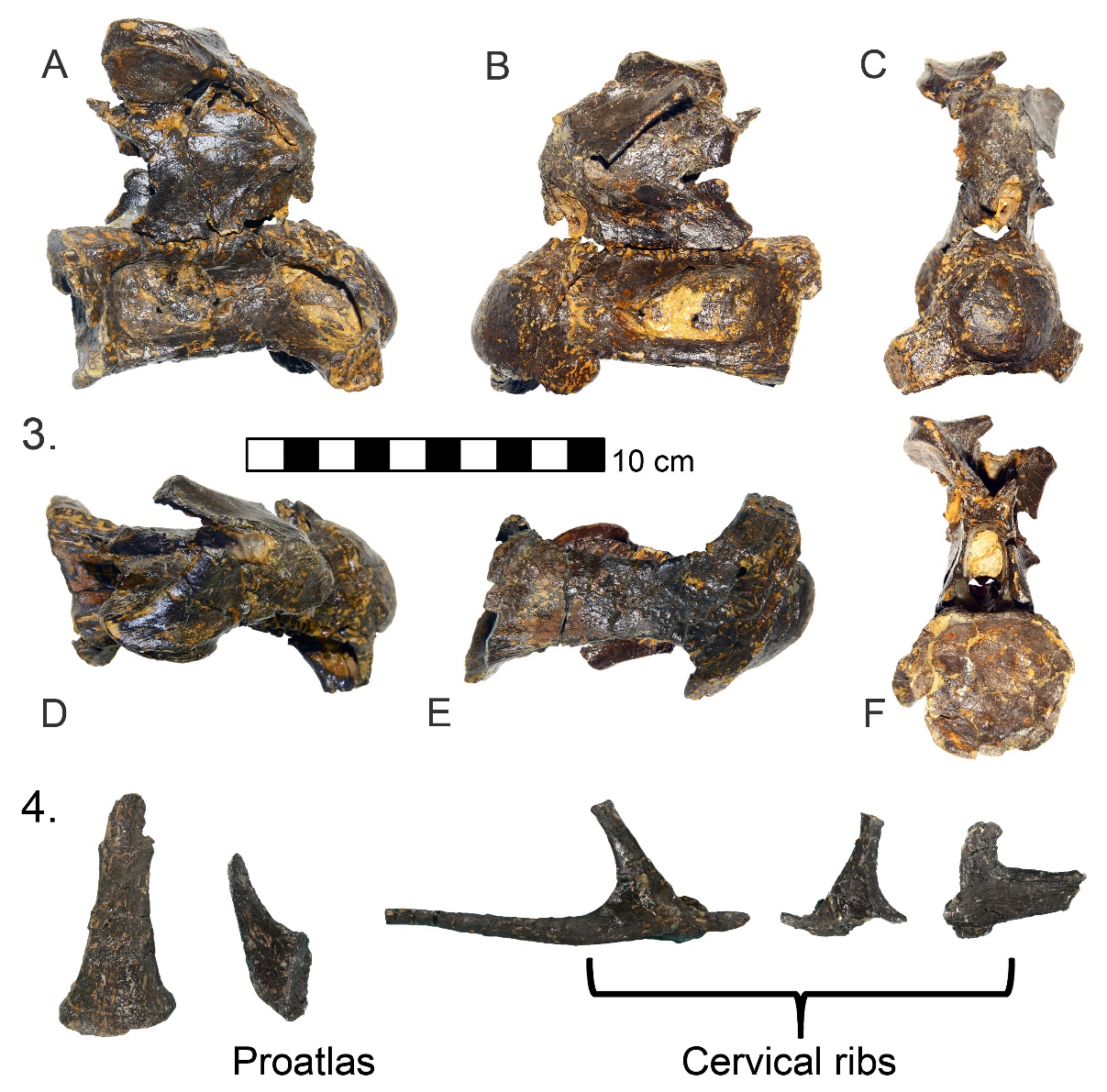


S.I. Fig. 2


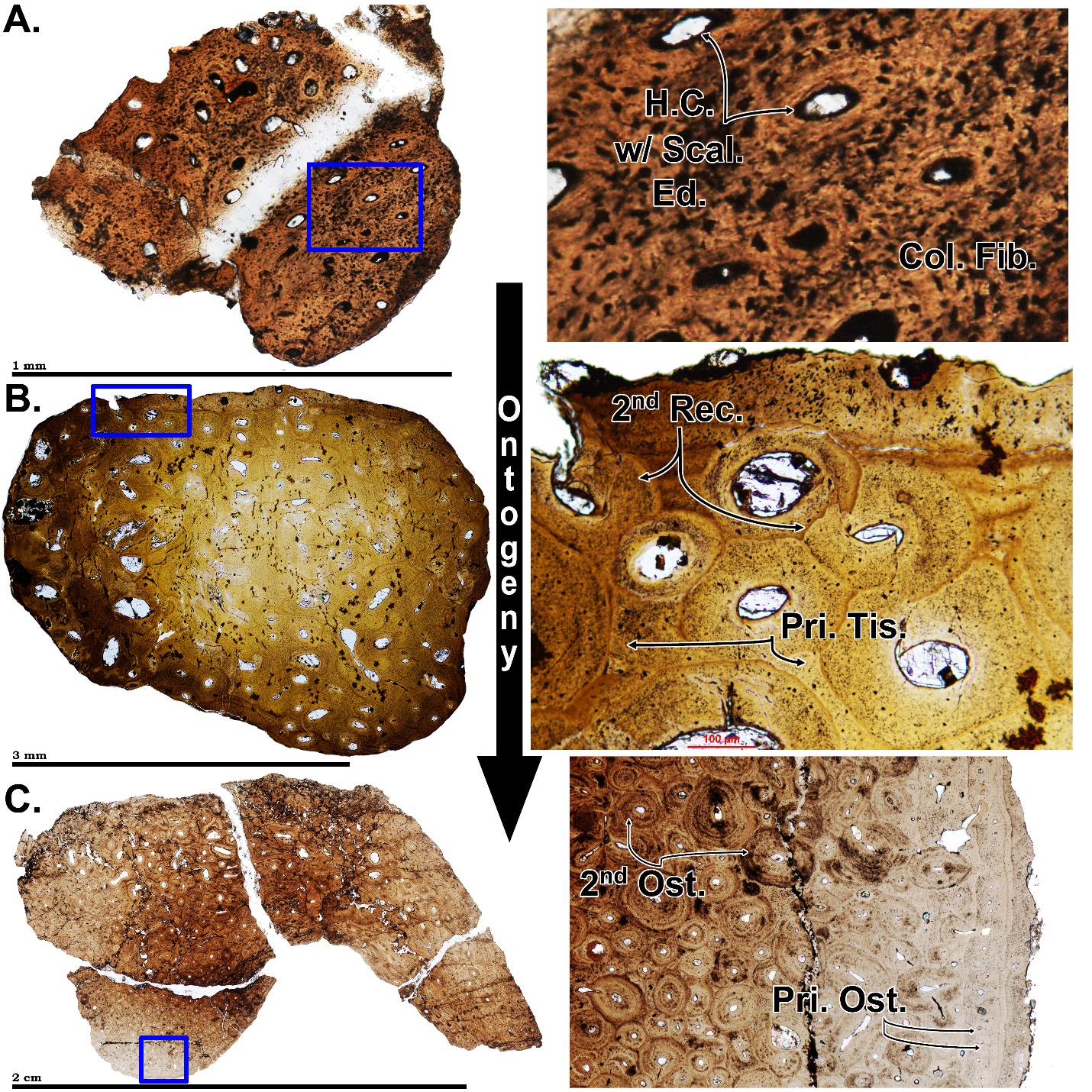


S.I. Fig. 3


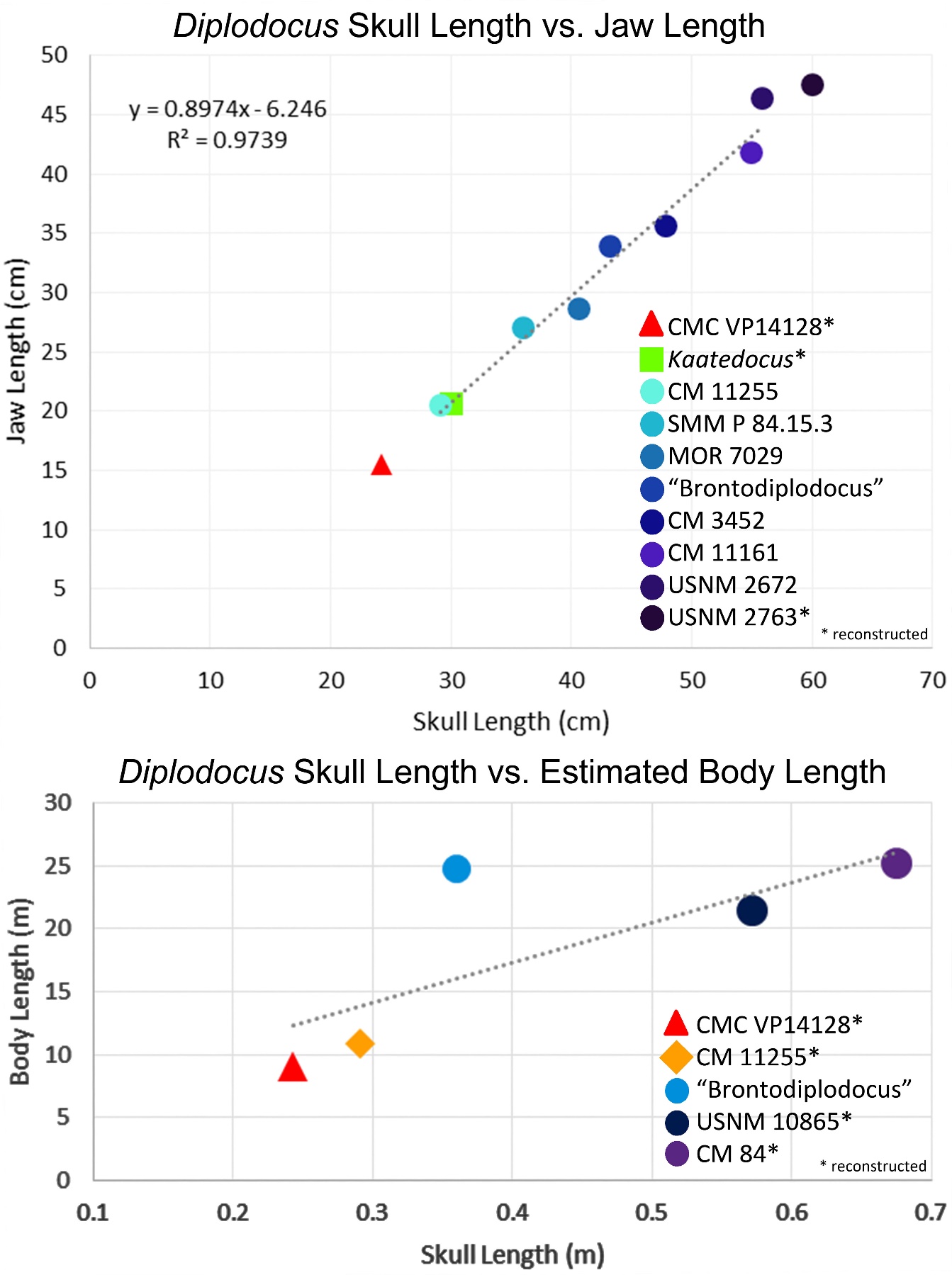


S.I. Fig. 4


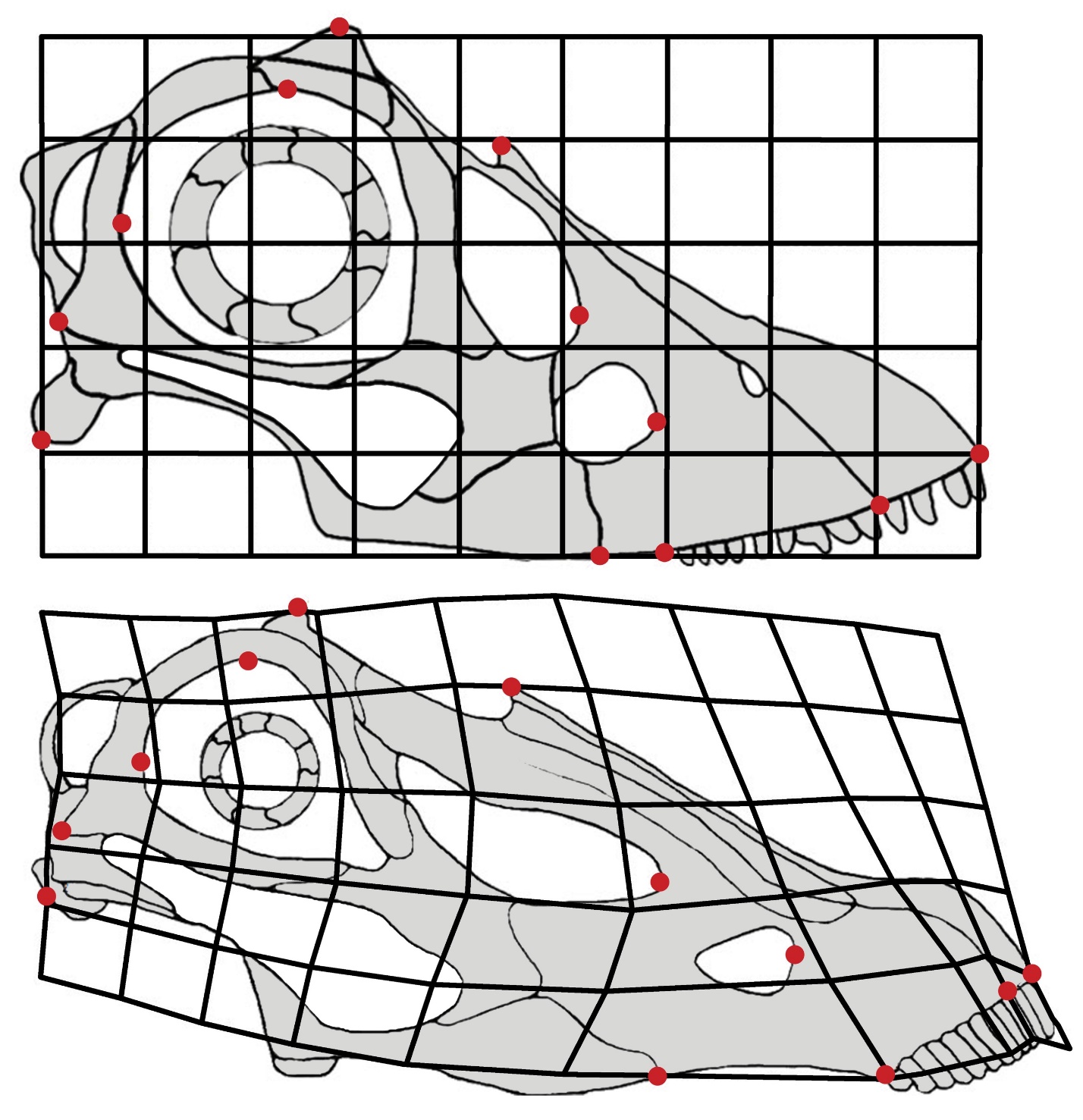


S.I. Fig. 5


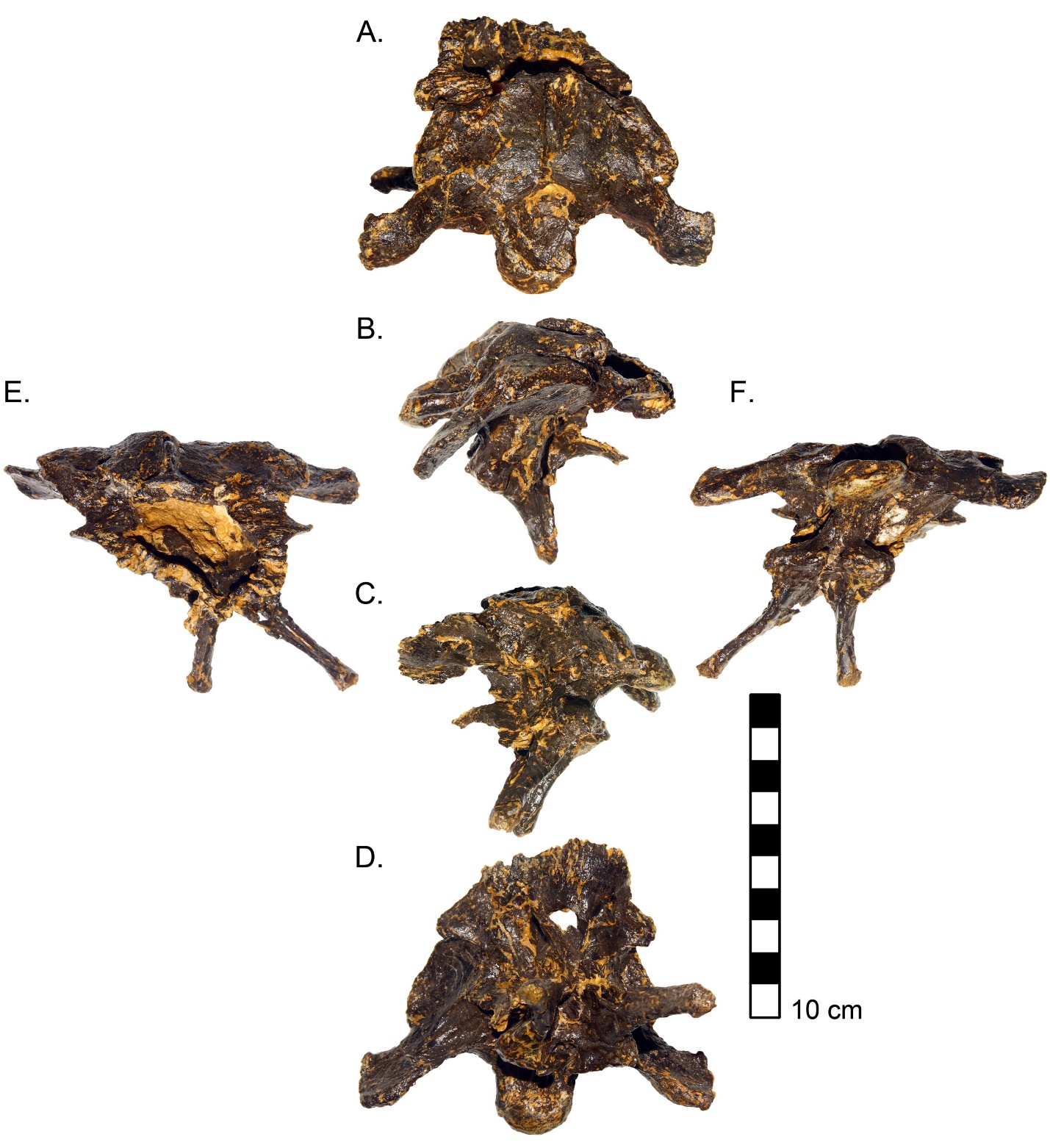


S.I. Fig. 6


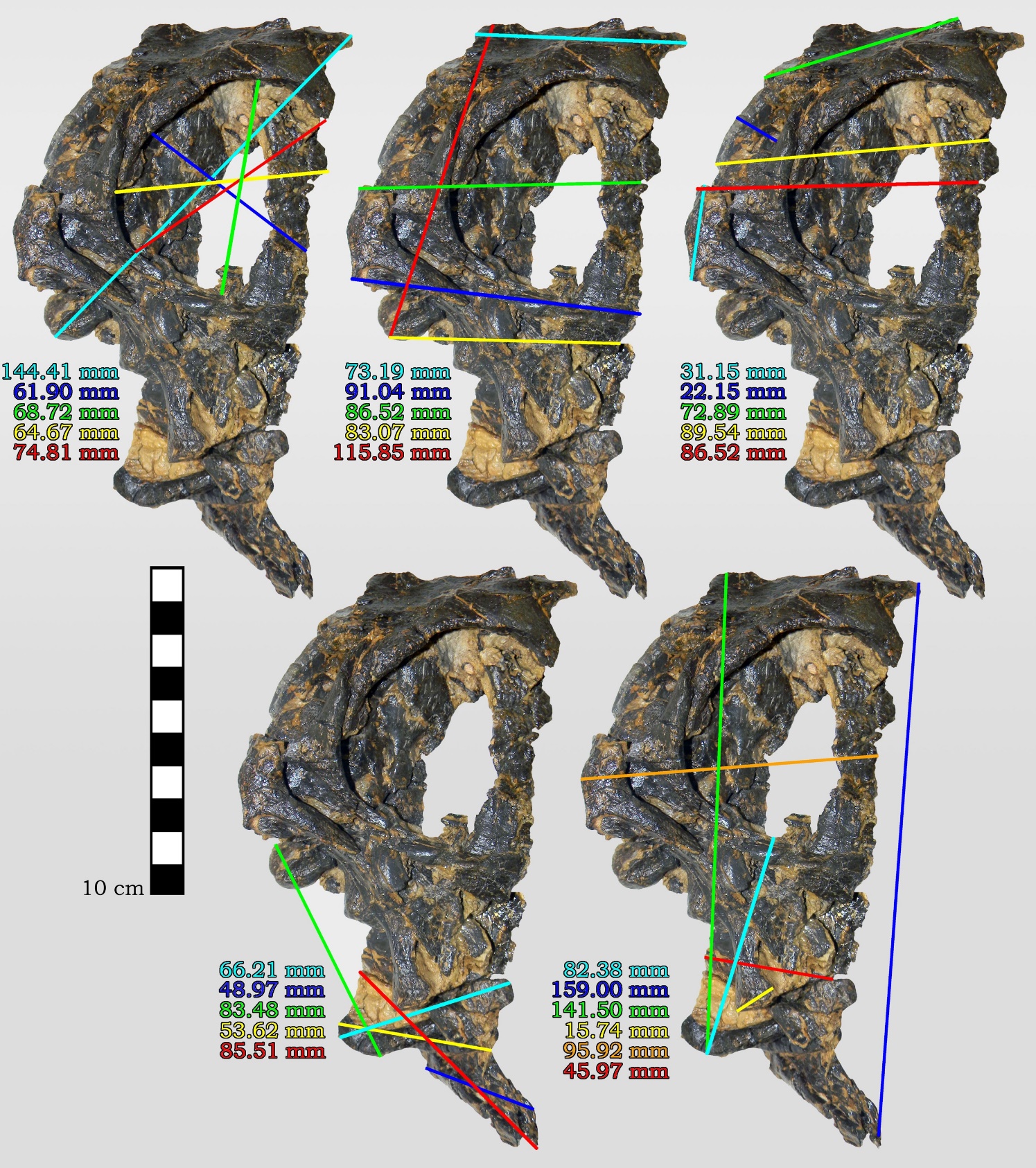


Measurement Fig. 1


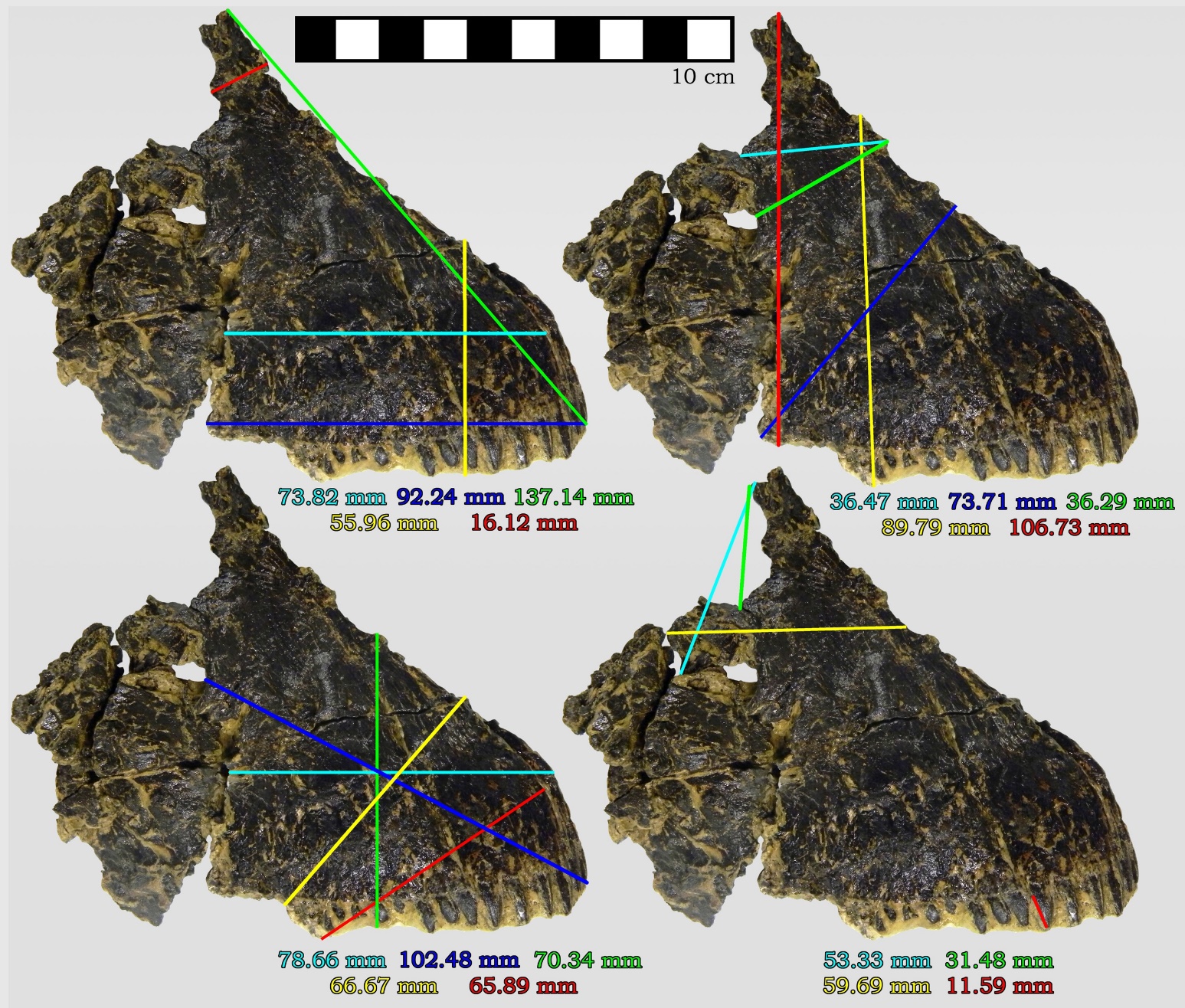


Measurement Fig. 2


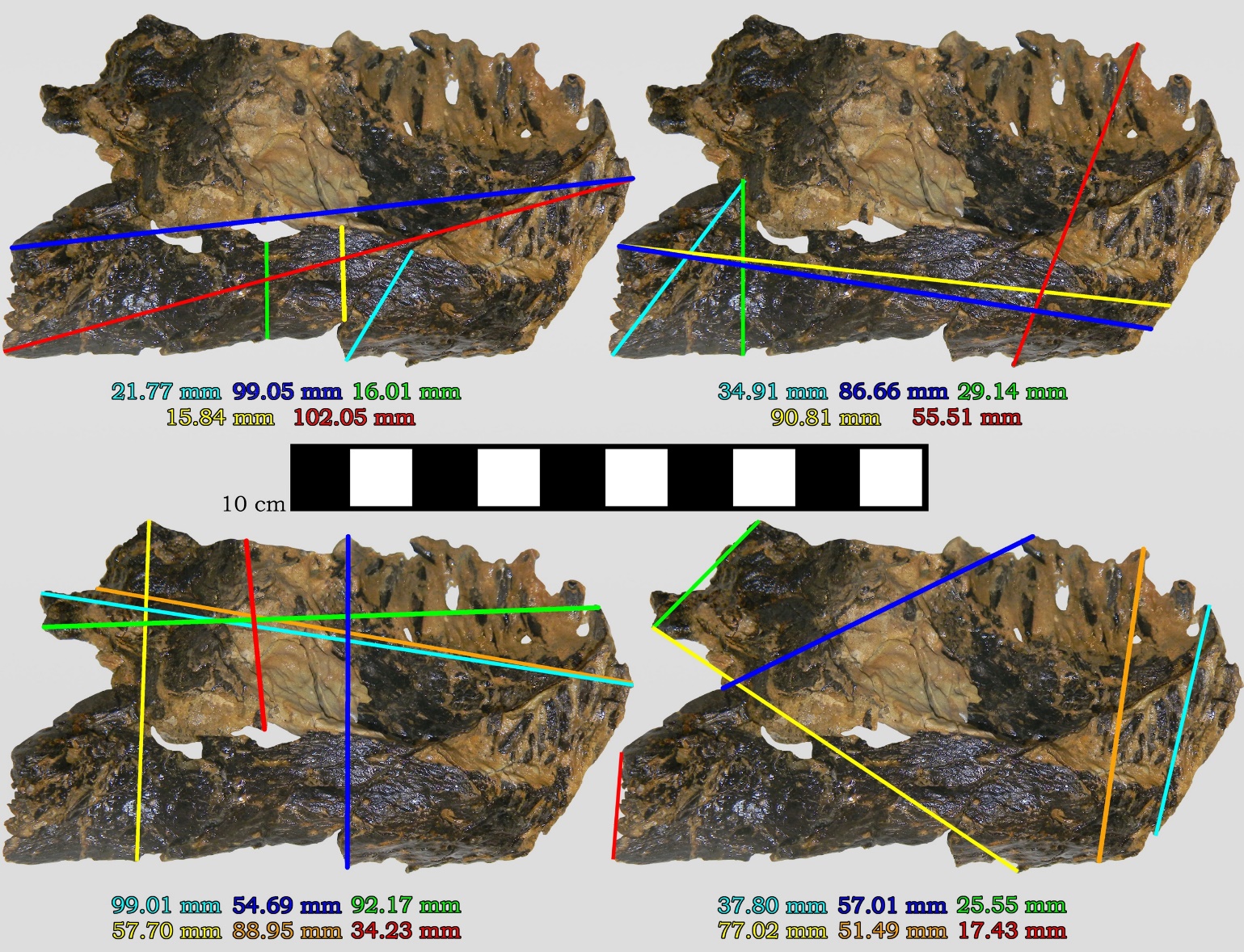


Measurement Fig. 3
