# Supplementary material for: The Smallest Diplodocid Skull Reveals Cranial Ontogeny and Growth-Related Dietary Changes in the Largest Dinosaurs
Source: Sci Rep. 2018 Oct 11;8:14341. doi: 10.1038/s41598-018-32620-x (PMC6181913; doi:10.1038/s41598-018-32620-x)
Supplement: Supplementary file 3 [file 41598_2018_32620_MOESM4_ESM.pdf]

Generated with Agisoft PhotoScan by Jack Wilson, Museum of the Rockies
